# The Psychology of Shame: A Resilience Seminar for Medical Students

**DOI:** 10.15766/mep_2374-8265.11052

**Published:** 2020-12-24

**Authors:** William E. Bynum, Sebastian Uijtdehaage, Anthony R. Artino, James W. Fox

**Affiliations:** 1 Associate Professor, Department of Family Medicine and Community Health, Duke University School of Medicine; 2 Professor, Department of Medicine, Uniformed Services University; 3 Professor, Department of Health, Human Function, and Rehabilitation Sciences, George Washington University School of Medicine and Health Sciences; 4 Professor, Department of Pediatrics, Duke University School of Medicine

**Keywords:** Shame, Resilience, Emotion, Transition Periods, Wellness, Error, Reflection/Narrative Medicine, Well-Being/Mental Health

## Abstract

**Introduction:**

Shame is a powerful emotion that can cause emotional distress, impaired empathy, social isolation, and unprofessional behavior in medical learners. However, interventions to help learners constructively engage with shame are rare. This module educated medical students about shame, guided them through an exploration of their shame experiences, and facilitated development of shame resilience.

**Methods:**

In this 2-hour workshop, clinical-year medical students were guided through the psychology of shame through didactic slides. Next, a small panel of volunteer students, recruited and coached prior to the workshop, shared reflections on the content, including their shame experiences during medical school. This was followed by didactic slides outlining strategies to promote shame resilience. Participants then broke into faculty-led small groups to discuss session content. The module included a small-group facilitator guide for leading discussions on shame, didactic slides, discussion prompts, an evaluation tool, and a film entitled *The Shame Conversation* that was created after the initial workshop.

**Results:**

A retrospective pre/postsurvey revealed statistically significant increases in: (1) importance ascribed to identifying shame in one's self or colleagues, (2) confidence in one's ability to recover from a shame reaction, and (3) comfort in reaching out to others when shame occurs. Analysis of open-ended questions showed that students felt the seminar would enhance future resilience by helping them identify and normalize shame, distinguish shame from guilt, and reach out to others for help.

**Discussion:**

This workshop appears to prepare students to more constructively engage with shame when it occurs in medical training.

## Educational Objectives

By the end of the seminar, learners will be able to:

1. Define shame and guilt and distinguish them from one another.
2. Identify shame when it occurs, either in themselves or in others, while learning medicine.
3. List specific strategies to build resilience through constructive engagement with shame.
4. Report increased willingness to reach out to others should they experience shame in the future.
5. Report increased confidence in their ability to recover from a shame reaction and to help others recover from a shame reaction.

## Introduction

In response to Liaison Committee on Medical Education requirements to foster learner wellness, medical schools are tasked with creating meaningful programs that promote resilience in medical students.^1^ Existing programs have focused on strategies such as mindfulness, enhanced self-care, and changes to organizational structure to advance learner wellness.^2–4^ Few published strategies have focused on promoting emotional resilience in learners, and only one published innovation has directly addressed shame in medical students.^5^ Notably, the authors of this paper reported inadvertently inducing shame in participating students, a workshop outcome that highlights the challenge of directly addressing this oft-stigmatized topic.^5^

Shame is a normal, negative self-conscious emotion that occurs when an individual engages in a self-evaluation and attributes a triggering event (e.g., a medical error, test failure) to a global deficiency of the self.^6,7^ The shamed individual feels flawed, deficient, and/or unworthy and is unable to separate specific actions from the global self.^6,7^ Studies from psychology show that shame often leads to withdrawal, isolation, and hiding,^6^ and is associated with depression, anxiety, posttraumatic stress disorder, and impaired empathy, among other negative outcomes.^8–11^ In a qualitative study on shame in medical residents, participants described shame as a potentially debilitating emotion that could lead to similar negative outcomes, including depressive feelings, isolation and withdrawal, unprofessional behavior, and impaired empathy.^12^ Factors both internal and external to residents triggered and/or contributed to their shame experiences. Internal factors such as perfectionism, fear of judgment, and a tendency to compare to others contributed to shame, and environmental forces including mistreatment, psychologically unsafe environments, and harsh remediation processes all contributed to shame in resident participants.^12^

Guilt is a related but distinct self-conscious emotion. Guilt occurs when an individual attributes a triggering event to a specific action or circumstance that can be modified, rather than the global self.^13,14^ Guilt often prompts reparative actions and efforts to undo potential harm.^13,14^ As such, it is considered a more constructive emotion that promotes engagement and growth. Leveraging guilt following a triggering event (i.e., by focusing on specific actions and not the generalized self) may be one specific way to promote shame resilience in medical learners, which has been described as the ability to proactively confront shame in a way that enhances one's self-concept, increases a sense of power and control, and restores one's sense of interpersonal connection and social standing.^8^

Shame resilience can be thought of as the ability to proactively and authentically engage with shame in a manner that facilitates healing, recovery, and growth. There are no published data on how medical learners develop resilience to shame; however, existing theory in psychology and sociology offer direction. In her grounded theory study of shame resilience in adults, Van Vliet proposed that individuals recover from shame through a process of self-reconstruction that encompasses connecting, refocusing, accepting, understanding, and resisting.^8^ Sociologist Brené Brown has identified four basic components of shame resilience: recognizing shame and understanding its triggers, practicing critical awareness of the influences leading to shame, reaching out to others, and naming shame when it occurs.^15^ In both theories, shame resilience is thought of as active engagement with shame, rather than avoidance and withdrawal.

The gap in our knowledge of how best to address and educate about shame—ultimately in the hopes of promoting shame resilience—is significant. Medical school presents numerous challenges that may provoke shame responses in medical students, including challenging team dynamics, exposure of knowledge deficits, unexpected failures, and frequent transition periods.^12^ Furthermore, environmental influences such as suboptimal learning environments, mistreatment from others, lack of vulnerability demonstrated by superiors, intense competition, and hierarchical pressures may contribute to shame or impede a learner's ability to reach out for help if it occurs.^16,17^ Given the destructive potential of shame and its links to negative outcomes in medical learners, educational innovations are needed to enable medical students to proactively and constructively engage with this emotion.

Thus, this seminar sought to introduce students to the construct of shame and utilize open discussion, within a safe environment, to promote the development of shame resilience. In this publication, we present the framework and resources needed to conduct and evaluate this discussion-based shame resilience seminar for medical students. We previously published the description and evaluation of a workshop on shame resilience.^18^ With this project, we provided more details on the development of the workshop and the resources needed for educators to implement it in other settings.

## Methods

### Curricular Context

We conducted this 2-hour seminar, entitled “A Resilience Seminar: Recognizing and Constructively Engaging with Shame,” as a part of a longitudinal course called Clinical Skills Foundation (CSF) that spans the first two years at Duke University School of Medicine. The seminar was held in February of the clinical year of medical school, which occurs in the second year at Duke University and consists of the clinical clerkships. During the clinical year, CSF sessions are held weekly for 4 consecutive weeks in every 8-week clerkship period and adhere to the following format: a 30–60 minute large-group session followed by 60–90 minute small-group breakouts. The student composition of the small groups remains the same across the 2 years, and each small group is comprised of seven to eight students and two group leaders (one faculty member and one senior-level medical student). CSF small-group leaders, including faculty and senior-level medical students, are chosen through a competitive application process. Each CSF session covers a specific topic, and the longitudinal small groups provide a familiar and safe environment for students to share their feelings and experiences related to the topic.

### Development

Prior to executing the seminar, we developed local needs and assets assessments by meeting with student leaders, advisory deans, and CSF course directors. Student leaders provided suggestions and context (e.g., ways that shame might manifest in the local learning environment) to optimize the relevance of the seminar content. Two student leaders volunteered to speak during the large-group session about their own experiences with shame during medical school. Advisory deans provided information about ongoing resilience initiatives that helped us align the seminar with existing resources. Course directors provided historical input about successful and unsuccessful approaches to leading a CSF session that helped us optimize the effectiveness of our approach. For example, course directors advised us to limit time spent on didactic slides, not portray shame as an inevitable experience, not require students to share in front of the large group, and save plenty of time for small-group discussion.

We also created a five-page small-group facilitator guide outlining the seminar goals and objectives, session timeline, tips for navigating sensitive conversations about shame, an overview of the psychology of shame and guilt, an overview of relevant findings from new research on shame in medical learners, strategies for promoting shame resilience in medical education, and suggested prompts for small-group discussion (Appendix A). We provided the guide to small-group leaders 1 week prior to the seminar to review ahead of time.

### Implementation

Of note, there was no required prereading for students because we wanted to introduce the topic in person before having them engage in their own reading and reflection.

Thirty minutes prior to the seminar start time, we met with small-group leaders and fielded any questions and concerns about the seminar content. Numerous leaders expressed concerns about leading discussions on a personal and sensitive topic like shame. We discussed these concerns as a group and suggested strategies for navigating sensitive discussions and learner distress. We developed these strategies with assistance from upper-level medical students to maximize relevance and applicability (Appendix A).

We executed the seminar in two parts: a large-group session and small-group break-out discussions. The large-group session consisted of four basic activities:

1. The session facilitator opened the presentation by sharing two personal shame stories during his time as a medical learner: a shame reaction following a significant medical error and a shame reaction occurring during transition period on a trauma rotation. The facilitator used slides to visually support his storytelling, which was told from the first-person point of view. This portion lasted approximately 10 minutes. Note: In the time since the workshop, we created a film entitled *The Shame Conversation* that depicts narrative accounts of shame from diverse health care professionals. This film can be used as a substitute for—or adjunct to—sharing a personal narrative of shame, which can be challenging. The film (Appendix B) can be also accessed for free at www.theshameconvo.com.
2. Next, the facilitator introduced the psychology of shame and guilt based on Tracy and Robins’ theory of self-conscious emotion.^19^ Using PowerPoint slides as a visual reference (Appendix C), he walked students through the four basic appraisals and attributions that give rise to—and differentiate—shame and guilt. The PowerPoint presentation continued with a brief overview of relevant findings from a recent qualitative assessment of shame in medical residents.^12^ The facilitator concluded the PowerPoint portion of the large-group session with a brief overview of four basic components of shame resilience from Brené Brown.^15^ He articulated specific strategies for developing shame resilience that have emerged through an ongoing program of research on shame in medical learners.^12,20^ This portion lasted approximately 25 minutes.
3. Third, a panel of two third-year students provided their own shame experiences as medical students. The students did not utilize visual aids, and each student spoke for 5–7 minutes. They shared feelings of inadequacy compared to fellow classmates, struggles with imposter syndrome, difficulty sharing emotions with colleagues and supervisors, and personal strategies for constructively dealing with shame. This portion lasted approximately 12 minutes.
4. The large-group session concluded with a brief question and answer session with the session facilitator and student speakers. This portion lasted approximately 8 minutes.

The small-group session consisted of students breaking into their respective longitudinal small groups for discussions facilitated by the small-group leaders. We provided two suggested prompts to small-group leaders to help guide discussions (Appendix D). We advised small-group leaders to spend about half of the allotted time (approximately 30 minutes) on each question.

1. Prompt 1**:** Explore and discuss your reactions to the information presented in the seminar. Consider sharing any experiences you have had with shame or guilt as a medical student (or that you have encountered in others). How did you feel and what contributed to your feelings?
2. Prompt 2: Brainstorm and discuss specific strategies that you have utilized or would utilize to adopt a shame-resilient approach to learning medicine. Consider not only individual strategies but also ways you might positively influence the learning environment to promote open, safe sharing of shame experiences.

Small-group sessions lasted for 60–90 minutes depending on the group. Importantly we created outlets for both students and small-group leaders to report any distress, concerning discussion, or unexpected occurrences that arose during the small-group discussions.

### Assessment

At the end of the small-group session, students were asked to complete a voluntary, retrospective pre/postsurvey evaluating the seminar effectiveness (Appendix E). The survey assessed students’ satisfaction, likelihood of implementing seminar content, and changes in attitudes, confidence, and willingness to reach out for help (levels I and II of Kirkpatrick's pyramid).^21^ We developed the survey to evaluate the session objectives, and we utilized literature on shame from psychology^13^ and medical education^12^ to inform item selection. Two authors of the survey (Anthony R. Artino and Sebastian Uijtdehaage) are content experts in survey design, and they contributed heavily to its development. We conducted informal pilot testing with colleagues to ensure that the wording was clear and to gauge input about the length. We then utilized this input to create a final version of the survey.

We asked small-group leaders (including faculty and student leaders) to complete a separate evaluation after the seminar concluded (Appendix E) to assess for the presence of student distress as a result of the seminar and to identify ways to improve the seminar in the future. We asked group leaders the following questions: “Did any students report distress?,” “What was the nature of the distress?,” and “What can be done differently in the future to avoid risk of similar distress?” Small-group leaders were prepared to reach out to any students expressing distress after the seminar, and the school of medicine advisory deans were on standby to support distressed students. A psychologist who routinely provided counseling to students was also aware of the seminar. Small-group leaders were encouraged to report any unexpected occurrences or concerning discussions to the CSF course directors, who would then communicate the concerns to the appropriate parties depending on the nature of the situation.

The Duke University Institutional Review Board determined that this study was exempt from full review.

## Results

In total, 113 second-year medical students and 31 small-group leaders participated in the seminar. Of the 113 students in attendance, 80 attempted the survey (71% response rate): 62 (55%) completed the entire survey, and 18 failed to complete both sections of retrospective the pre/post questions due to a formatting glitch in the web-based survey. The results of the evaluation are listed in the Table.

**Table.**
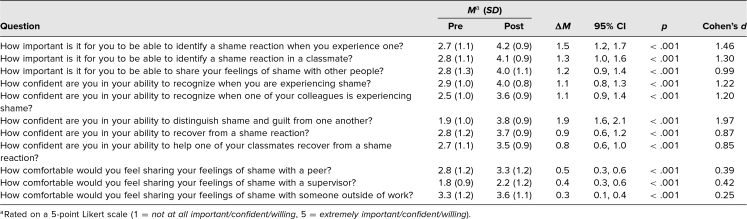
Results From the Retrospective Pre/Postsurvey of Second-Year Medical Students (*N* = 113)

These data showed statistically significant increases in: the level of importance students ascribed to identifying a shame reaction in themselves (*M*_pre_ = 2.7, *M*_post_ = 4.2, *p* < .001, Cohen's *d* = 1.46) and in sharing their feelings of shame with other people (*M*_pre_ = 2.8, *M*_post_ = 4.1, *p* < .001, Cohen's *d* = 1.30); students’ confidence in their ability to recognize shame in themselves (*M*_pre_ = 2.9, *M*_post_ = 4.0, *p* < .001, Cohen's *d* = 1.22) and others (*M*_pre_ = 2.5, *M*_post_ = 3.6, *p* < .001, Cohen's *d* = 1.20); distinguish shame from guilt (*M*_pre_ = 1.9, *M*_post_ = 3.8, *p* < .001, Cohen's *d* = 1.97); recover from a shame reaction (*M*_pre_ = 2.8, *M*_post_ = 3.7, *p* < .001, Cohen's *d* = 0.87); and students’ willingness to reach out to others when shame occurs. The effect sizes for the questions related to student attitudes (e.g., willingness, importance, confidence) were large, whereas the effect sizes of the questions related to reaching out to others (e.g., comfort sharing feelings of shame) were relatively small.

We performed inductive thematic analysis on two free-response questions: “What worked well in the large-group session?” and “How will participation in the seminar influence your resilience as a medical learner, if at all?” In regard to what worked well in the large-group session, our analysis revealed that the personal stories and vulnerability displayed by the speaker and student panel were effective at normalizing shame and instilling psychological safety for small-group discussions. Multiple students reported connecting with—and being most impacted by—their classmates’ stories. Two primary themes emerged from our analysis of students’ reflections on how the seminar will influence their future resilience: (1) the power of identifying and normalizing shame, and (2) the importance of reaching out to others. Students reported feeling that the ability to recognize and normalize shame, both in themselves and their peers, would enable them to build resilience and actively overcome shame in the future. In particular, multiple students reported appreciating their newfound ability to differentiate shame from guilt, including one who felt the seminar would “make me more sensitive to recognizing shame and guilt in myself and my peers.” Another student similarly reflected, “This [seminar] was extremely helpful in assisting me to label shame, recognize it in myself, and cause me to consider the specific and changeable features of what I am feeling.”

Multiple students also reported appreciation for the value of peer support, willingness to rely on peers when feeling shame, and commitment to supporting peers struggling with shame. One student reflected that the seminar “reinforced the importance of sharing with classmates,” a sentiment shared by another student who said, “[The seminar] will make me appreciate my peers and the support they can offer, as well as identifying my residents and advisors as ‘human’ who also feel the same sense of inadequacy at some point in their careers.”

Of small-group leaders, 14 (response rate 45%) completed the survey. Four small-group leaders (28%) reported student distress during their small groups. This distress manifested as tearfulness, feeling that the topic “hits very close to home,” and intense emotions due to the relevance of the topic. Group leaders reported that these reactions seemed productive, appropriate, and not overly distressing to students. These group leaders did not identify a need to change the format or content of the seminar in the future. Other group leaders suggested reducing the amount of total time spent in the large-group discussion—particularly the didactic portion—to allow even greater time for small-group discussion. Follow-up communication with advisory deans, course directors, and medical school leadership revealed no reports of student distress in the 2 months following the seminar, though unreported student distress may have occurred.

## Discussion

In this reproducible, low-resource seminar, we addressed a sensitive and difficult topic—personal shame—in a way that appears to have enhanced students’ attitudes about shame, confidence in identifying and recovering from shame, and willingness to reach out to others when they experience shame. Importantly, there is no evidence that our approach caused significant emotional distress in student participants, as has been reported by others.^5^

Numerous aspects of our seminar seemed to enable its positive outcomes and avoid learner distress. First, we used personal narrative to develop an emotional connection to the topic, model vulnerability, and establish an environment of psychological safety for subsequent small-group discussions. Based on feedback from students, having both students and faculty share shame stories was particularly effective. Second, combining personal stories with a primer on the psychology of shame and an overview of research in medical learners enhanced credibility and depicted shame as a common human experience in medical learners. Third, introducing the topic in a large group setting allowed students to reflect on their experiences prior to engaging in discussions in longitudinal small groups. Students had the opportunity to ask questions in the large group, but they were not prompted to share their experiences in this open forum. Fourth, the longitudinal nature of the small groups was critical to the seminar's apparent success, as the trust and relationships that had formed over time enabled open discussions about a sensitive topic. If longitudinal small groups are not feasible, efforts should be made to ensure a psychologically safe, trusting, and confidential environment in which these discussions can be held. Finally, by providing faculty development prior to the seminar, group leaders were better equipped to navigate these potentially sensitive topics and, for many, to share their own shame stories with students. As part of this faculty development, we provided group leaders with a list of strategies to navigate sensitive discussions such as these (Appendix A).

It is worth noting that the group discussions may be a meaningful opportunity to discuss the relationship between shame and underrepresentation. At the time of this seminar, our data had not pointed to such a relationship; however, in the time since, research on shame in medical students^20^ has helped us identify underrepresentation as a strong potential contributor to shame reactions. According to this study, being or feeling underrepresented in the learning environment can contribute to students’ feelings of inadequacy, impaired belonging, imposter syndrome, and intense pressure to prove their worthiness to be in medical school. Multiple participants acknowledged underrepresentation due to their race/ethnicity, sexual orientation, gender identity, hometown, academic pedigree, and religious beliefs.^20^ It is also likely—but currently unknown—that men, women, and those conforming to other gender identities experience shame differently. Thus, within a trusted and safe small-group setting, it may be worthwhile to explore students’ experiences of being underrepresented and how this influences their self-evaluation and interactions within the learning environment. Importantly, these discussions must take place in a respectful, accepting, and safe environment, and faculty should ideally be trained and prepared to lead them. If these features are not present, this topic should be approached very carefully, if at all.

Additionally, this workshop has the potential to be adapted for use in diverse populations, including other medical learners (e.g., residents, advanced practitioner students, nursing students, and allied health students) as well as practicing clinicians. Shame is a normal—albeit challenging—human emotion, and experiences of shame in health care are not limited to only medical students. Using this workshop in other settings should be done alongside careful consideration about the unique individuals and contexts within that setting.

### Limitations

The evaluation of our seminar was limited by the fact that it was conducted immediately following the seminar and that it utilized only a retrospective pre/postsurvey. This left little time for emotional processing. It was thus possible that students’ attitudes and reactions changed over time and that effect sizes may have been smaller with a true pre/postsurvey design. Further, our evaluation assessed only students’ attitudes, intentions, and reactions to the seminar; whether the seminar led to actual changes in behavior, wellness, and/or resilience was not assessed.

Regarding the reproducibility of this seminar, a primary limitation may be the availability and willingness of a person (or persons) to publicly share shame experiences with a large group. We recruited two volunteers by emailing the student small-group leaders, four of whom expressed interest in sharing their experiences. We then met with these students to inform them about the workshop and assess their comfort with participating. Two students ultimately volunteered. This highlights the fact that shame is a difficult emotion to openly discuss, and finding individuals willing to be vulnerable in front of a large group may be a challenge. Normalizing shame, facilitating open and psychologically safe environments, and pointing to other instances in which people have shared their shame (e.g., this seminar, *The Shame Conversation* film, and videos created by Brené Brown) might increase individuals’ comfort and willingness to openly share their stories with a large group. We utilized each of these approaches during meetings with potential volunteers. If volunteers are not an option, *The Shame Conversation* film can be used instead.

A second limitation in reproducing our seminar may be the lack of availability of longitudinal small groups. This does not need to preclude attempts at the seminar, but if ad hoc small groups are formed, efforts should be taken to develop trust and psychological support. Group leaders should establish confidentiality, consider sharing their own shame stories to model vulnerability, and provide opportunities for students to share something personal about themselves prior to opening up about their shame experiences. Students should commit to respectful treatment of one another and should adopt open, accepting stances to the experiences of others. We recommend explicitly soliciting these commitments before starting the small group discussions.

### Future Directions

As a next step, we are developing an empirically derived, longitudinal shame resilience curriculum spanning the medical student years. We will utilize the same basic format described above, but will explore different aspects of the shame experience that are most relevant to students’ current level of training. This will be informed by our ongoing research into how medical students experience shame as well as input from current students. For example, an initial seminar might focus on imposter syndrome, belonging, and expectations of one's self. Later seminars might focus on shame during transition periods, shame in the clinical learning environment, and shame following a medical error.

As a part of this curriculum, we have conducted a second iteration of this seminar with clinical-year medical students at Duke (approximately 110 students). We adhered to the same structure outlined above—including having two students share their shame experiences with the class—but spent less time on the didactic portion, more time in small-group discussion, and more time preparing group leaders, with whom we met 1 week prior to the seminar instead of on the same day. We have also conducted two seminars on shame and medical error for fourth-year medical students 2 months prior to graduation (approximately 100 students in each seminar). We used a similar approach as that outlined above. A current resident provided a narrative account of shame triggered by a medical error followed by an exploration of the subsequent emotions, effects, and recovery strategies that can follow a medical error. Workshop evaluations yielded similar results as those presented above.

The potential implications of initiatives designed to promote resilience to shame are significant: it appears that shame underlies or contributes to major challenges affecting health care students and providers, including depression, burnout, impaired empathy, and unprofessional behavior. Compounding this issue is the potential for shame to induce hiding, isolation, and withdrawal. Thus, bringing shame into the open in an engaged, constructive manner is a critical first step to addressing its role in the well-being of health care learners. Providing a seminar in which students can, within a psychologically safe environment, learn about, identify, and reach out about their shame is a tangible way to achieve this goal.

## Appendices

Small Group Facilitator Guide.docxThe Shame Conversation Film.mp4Didactic Slides.pptxSmall Group Discussion Prompts.docxWorkshop Evaluations.docx
All appendices are peer reviewed as integral parts of the Original Publication.
