# Supplementary material for: The Psychology of Shame: A Resilience Seminar for Medical Students
Source: MedEdPORTAL. 2020 Dec 24;16:11052. doi: 10.15766/mep_2374-8265.11052 (PMC7780736; doi:10.15766/mep_2374-8265.11052)
Supplement: Supplementary file 1 — Small Group Facilitator Guide.docxThe Shame Conversation Film.mp4Didactic Slides.pptxSmall Group Discussion Prompts.docxWorkshop Evaluations.docx [file mep_2374-8265.11052-s001.zip › A. Small Group Facilitator Guide.docx]

**Addressing the Elephant in the Room**

A Shame Resilience Seminar for Medical Students

**
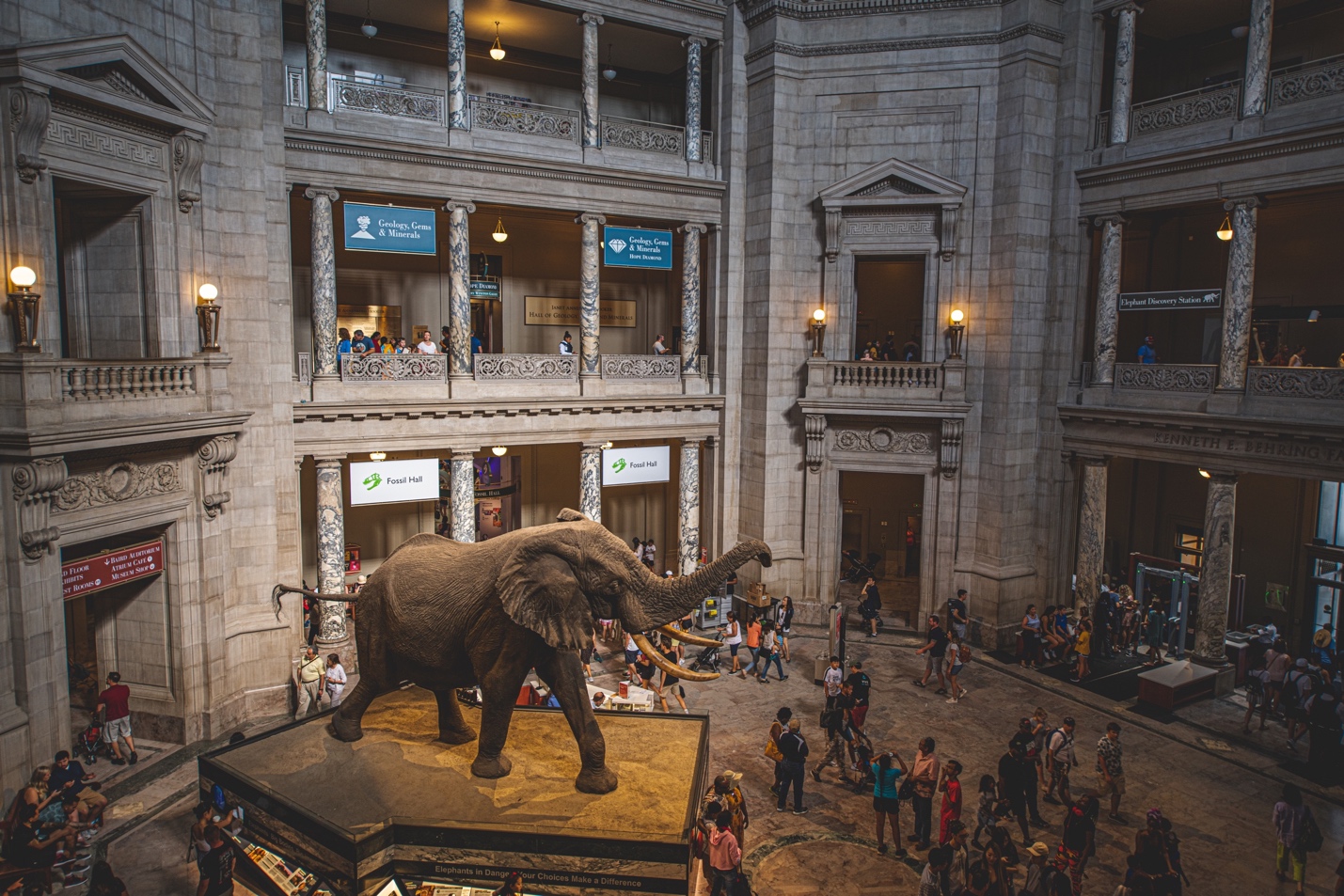
**

**Appendix A – Small Group Facilitator Guide**

Prepared by Will Bynum, MD

Associate Professor of Family Medicine

Duke University School of Medicine

william.e.bynum@duke.edu

Image by mana5280, retrieved from [www.unsplash.com](http://www.unsplash.com) on Nov 10, 2019.

Creative Commons license associated: <https://unsplash.com/license>

**What is shame?**

Shame is a self-conscious emotion that occurs when an individual assesses him/herself to be ***globally flawed*** or deficient in response to a transgression or failure to reach an expectation. Studies outside of medicine have revealed associations between shame and major psychological distress, including depression, anxiety, and suicidality, and shame often leads to a desire to hide, disengage, and isolate.

A self-evaluation is required for shame to occur. **Tracy & Robins’ theory of self-conscious emotions**^1,2^ outlines the appraisals and attributions through which a self-evaluation leads to shame, guilt, and two types of pride. A key appraisal is whether the individual’s current view of him/herself (as influenced by the event that triggered the self-evaluation, e.g. making an error) aligns with his/her ideal self. The ideal self is characterized by the standards and expectations a person has set for him/herself. The other critical appraisal is on what the individual places blame should he/she determine that his/her current and ideal selves do not align. If blame is placed on something global and unchanging (e.g. intellect, capability, attractiveness, etc) then shame occurs; if it is placed on something specific and changeable (e.g. level of effort expended, experience level, etc) then guilt occurs (see below).


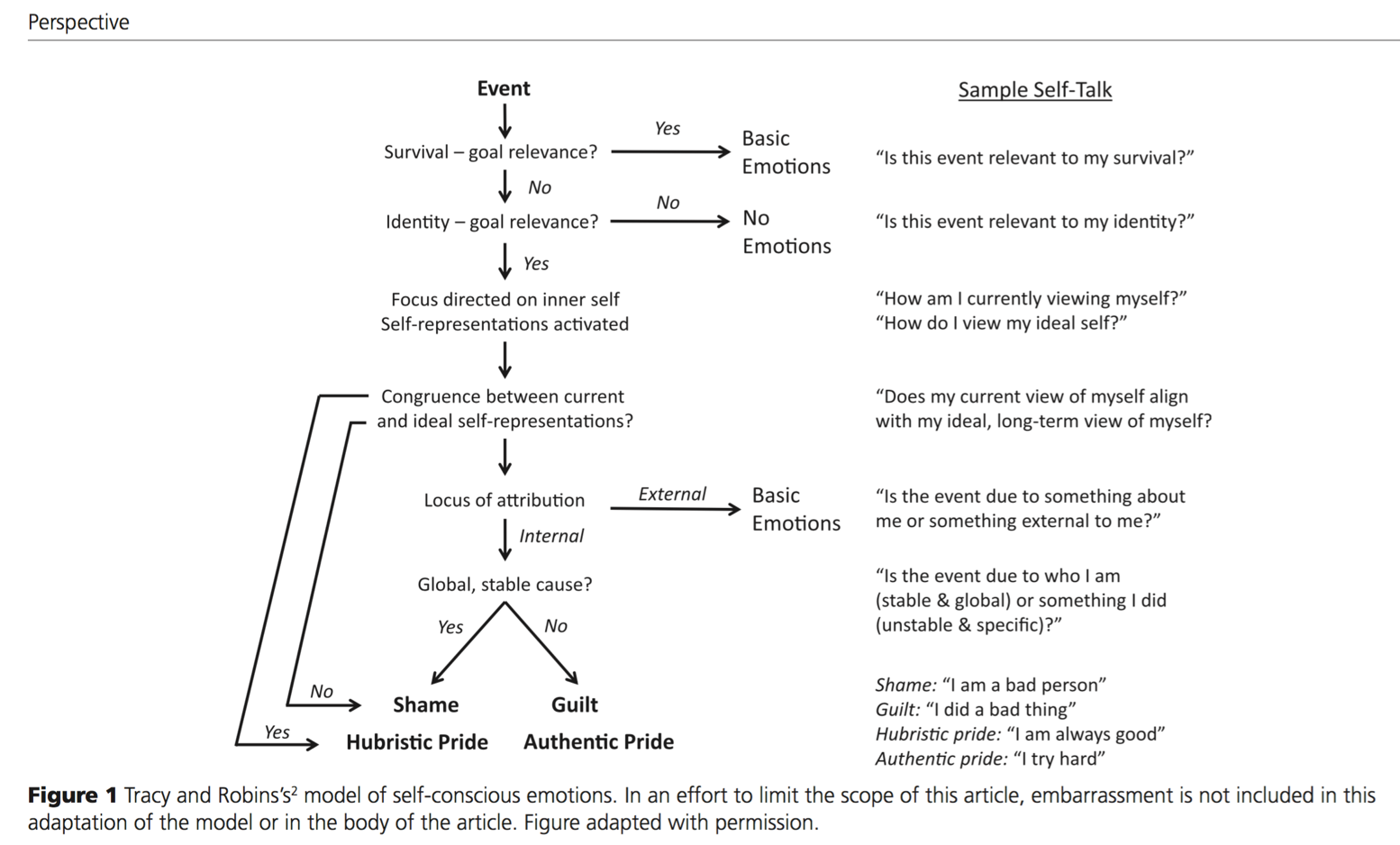


Figure from: Bynum WE, Artino AR, Jr. Who am I, and who do I strive to be? Applying a theory of self-conscious emotions to medical education. *Academic Medicine.* 2017;93(6):874-880. Used with permission.

**It is important to note that shame is a normal human emotion that can have pro-social functions: as a self-conscious emotion, it guides human behavior by “compelling us to do things that are socially valued and to avoid doing things that lead to social [dis]approbation.”^3(p. 194)^ In other words, shame may alert us to potential negative social consequences of behavior in order to maintain our social status or belonging in a group. However, enduring or intense shame can lead to negative outcomes, including social withdrawal, impaired belonging, poor physical or psychological health, unprofessional behavior, and impaired empathy.

*Thus, the message to students is NOT to avoid, ignore, or eliminate shame. Rather, the message to students is to be able to identify shame when it occurs and constructively engage with it to avoid the negative outcomes that occur with intense/enduring shame.*

**Understanding the differences between shame and guilt**

As mentioned above, shame and guilt are both self-conscious emotions that result from a self-evaluation. **Neither one feels good, but the outcomes and action tendencies are significantly different such that shame is generally considered a destructive emotion and guilt a constructive emotion.**

**
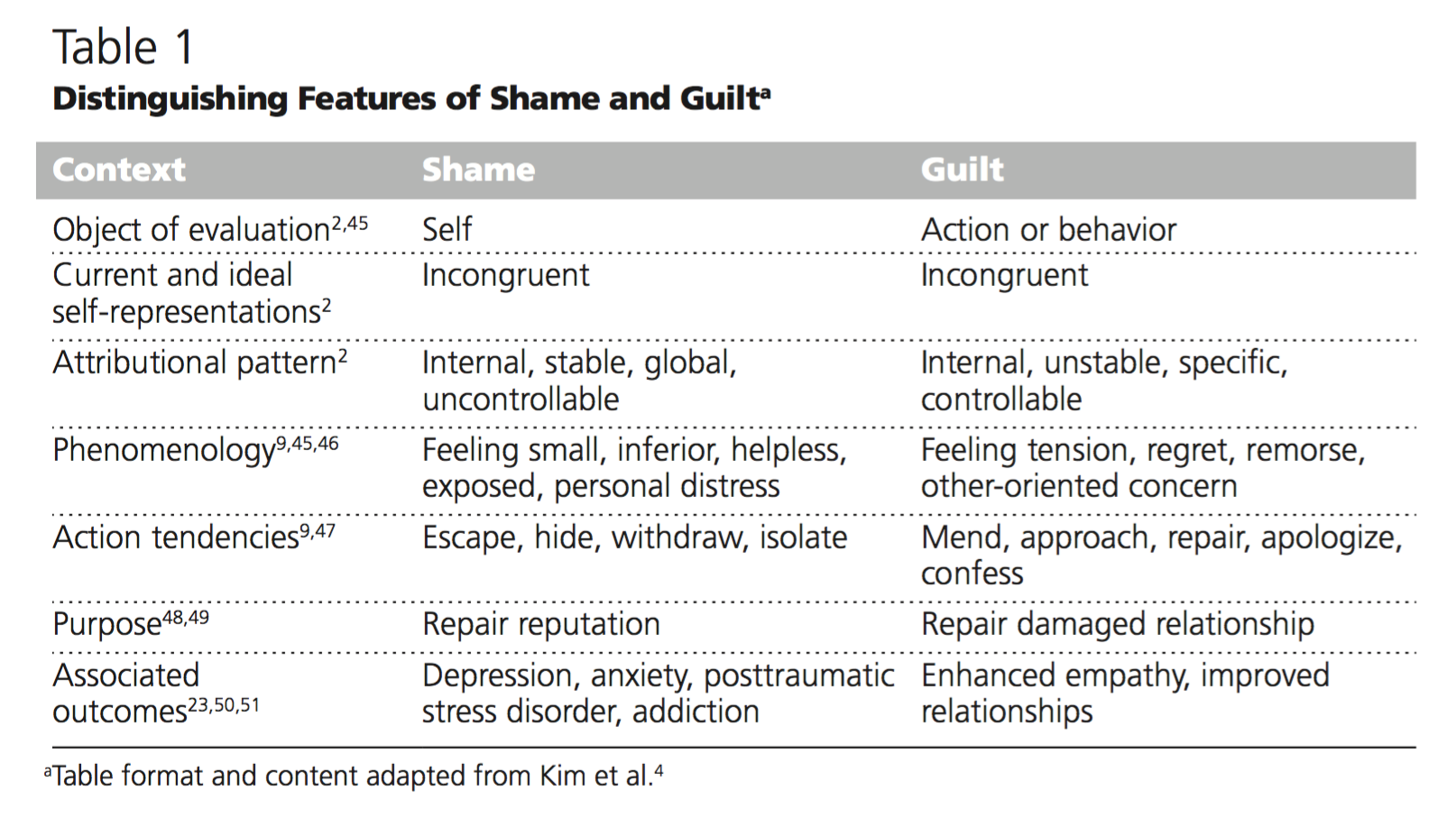
**

Figure from: Bynum WE, Artino AR, Jr. Who am I, and who do I strive to be? Applying a theory of self-conscious emotions to medical education. *Academic Medicine.* 2017;93(6):874-880. Used with permission.

**Shame in Transition Periods**

Based on our research^4^, we have labeled major shame reactions in medical learners “sentinel emotional events” that can be profound physical and emotional events and lead to damaging outcomes. In our study on shame in medical residents^4^, we identified transition periods (particularly early medical school, early clinical year of medical school, and intern year) to be high risk periods for shame. Many factors that contributed to participants’ shame converged during these periods. These factors are likely relevant to medical students:

- Perfectionism
- Comparisons to others
- Excessive focus on performance
- Fear of judgment
- Impaired belonging & imposter syndrome
- Skewed frame of reference (self-analysis through frames of reference discordant from contextual realities…a.k.a. overly harsh self-evaluations)

In sum, medical students may experience feelings of shame during transition periods (or anytime) due to the perception that they are performing at a lower level than everyone else, don’t belong, and might be judged by others. This risk may be heightened further if they overly rely on quantitative metrics of performance to validate self-worth and/or they possess perfectionist standards of performance that are unachievable and unrealistic. These may learners them to self-evaluate in an overly harsh manner and in a way that is not in line with objective reality: i.e. a learner who feels like “I’m terrible at medicine, I’m not good enough, and I don’t belong here” when they are simply going through the normal learning process, which includes lapses in performance and necessary mistakes.

Importantly, the environment seems to mediate/influence the tendency of these influences to cause shame, and specific components of the environment may induce it (e.g. a teacher who humiliates learners). Environments that promote competition, undermine belonging, mistreat learners, and impair a learner’s willingness/ability to reach out for help are likely to increase the potential for learners to experience enduring/damaging shame.

**Promoting Shame Resilience in Medical Education**

**Shame Resilience (our definition):** the ability to engage with shame in a manner that facilitates recovery, growth, and constructive engagement with the emotion and prevents the negative outcomes that can occur.

Brene Brown has identified four basic components of shame resilience.^5^

1) recognizing shame and understanding its triggers

2) practicing critical awareness of the influences leading to shame

3) reaching out to others and gaining strength by knowing we’re not experiencing shame alone,

4) speaking shame: “If we cultivate enough awareness about shame to name it and speak to it, we’ve basically cut it off at the knees.”

A few additional points to consider:

- Confronting our perfectionism can build shame resilience
- Proactively transitioning to a guilt response can facilitate recovery from shame
- Reaching out to others during a shame reaction is critical, and peer-to-peer support is an effective way for students to build shame resilient environments within their sphere of influence
- Self-esteem that is broadly defined and does not overly rely on performance (particularly as measured quantitatively) may engender shame resilient approaches to learning medicine

**Tips to effectively facilitate discussions about shame**

Shame can be a sensitive topic for many, and the natural tendency is to withhold discussing challenging shame experiences. However, we have found that many learners appreciate the opportunity to talk about their shame experiences ***if provided with a safe environment in which to share them and in which shame is normalized.***

- Consider sharing your own experience(s) with shame and/or strategies for constructively engaging with it when it occurs.
- Shame is normal! However, shame can be a very damaging emotion, particularly when it frequent, intense, and/or enduring. ***One of the primary messages from this seminar is not to avoid shame, but rather to constructively and proactively engage with it when it occurs.***
- Basic knowledge about the origins and differences between shame and guilt will be necessary to successfully facilitate a small group discussion. See info above + attached references, if needed.
- Based on feedback from students during the planning stages of the seminar, small group discussions would be most useful if they go beyond simply defining or lamenting the problem (e.g. shame in medical education) and focus on solutions and specific actions students can take within their “sphere of influence.”
- Based on feedback from students, care should be taken to focus on *both* the individual and environmental forces that lead to shame in medical education. It may be helpful to discuss ways that learning environments can be reformed to promote shame resilience in learners and how medical students, acting within their spheres of influence, can contribute to these environments.

**References**

1. Tracy JL, Robins RW. Putting the self into self-conscious emotions: a theoretical model. *Psychological Inquiry.* 2004;15(2):103-125.

2. Bynum WE, Artino AR, Jr. Who am I, and who do I strive to be? Applying a theory of self-conscious emotions to medical education. *Academic Medicine.* 2017;93(6):874-880.

3. Tracy JL, Robins RW. Self-Conscious Emotions: Where Self and Emotion Meet. In: Sedikides C, Spencer SJ, eds. *The Self in Social Psychology.* New York, NY: Psychology Press; 2007.

4. Bynum WE, Artino AR, Jr., Uijtdehaage S, Webb AMB, Varpio L. Sentinel emotional events: the nature, triggers, and effects of shame experiences in medical residents. *Academic Medicine.* 2019;94(1):85-93.

5. Brown B. *Daring greatly: How the courage to be vulnerable transforms the way we live, love, parent, and lead.* 1st ed. New York, NY: Penguin Group; 2012.
