# Supplementary material for: The Psychology of Shame: A Resilience Seminar for Medical Students
Source: MedEdPORTAL. 2020 Dec 24;16:11052. doi: 10.15766/mep_2374-8265.11052 (PMC7780736; doi:10.15766/mep_2374-8265.11052)
Supplement: Supplementary file 1 — Small Group Facilitator Guide.docxThe Shame Conversation Film.mp4Didactic Slides.pptxSmall Group Discussion Prompts.docxWorkshop Evaluations.docx [file mep_2374-8265.11052-s001.zip › C. Didactic Slides.pptx]

## Slide 1
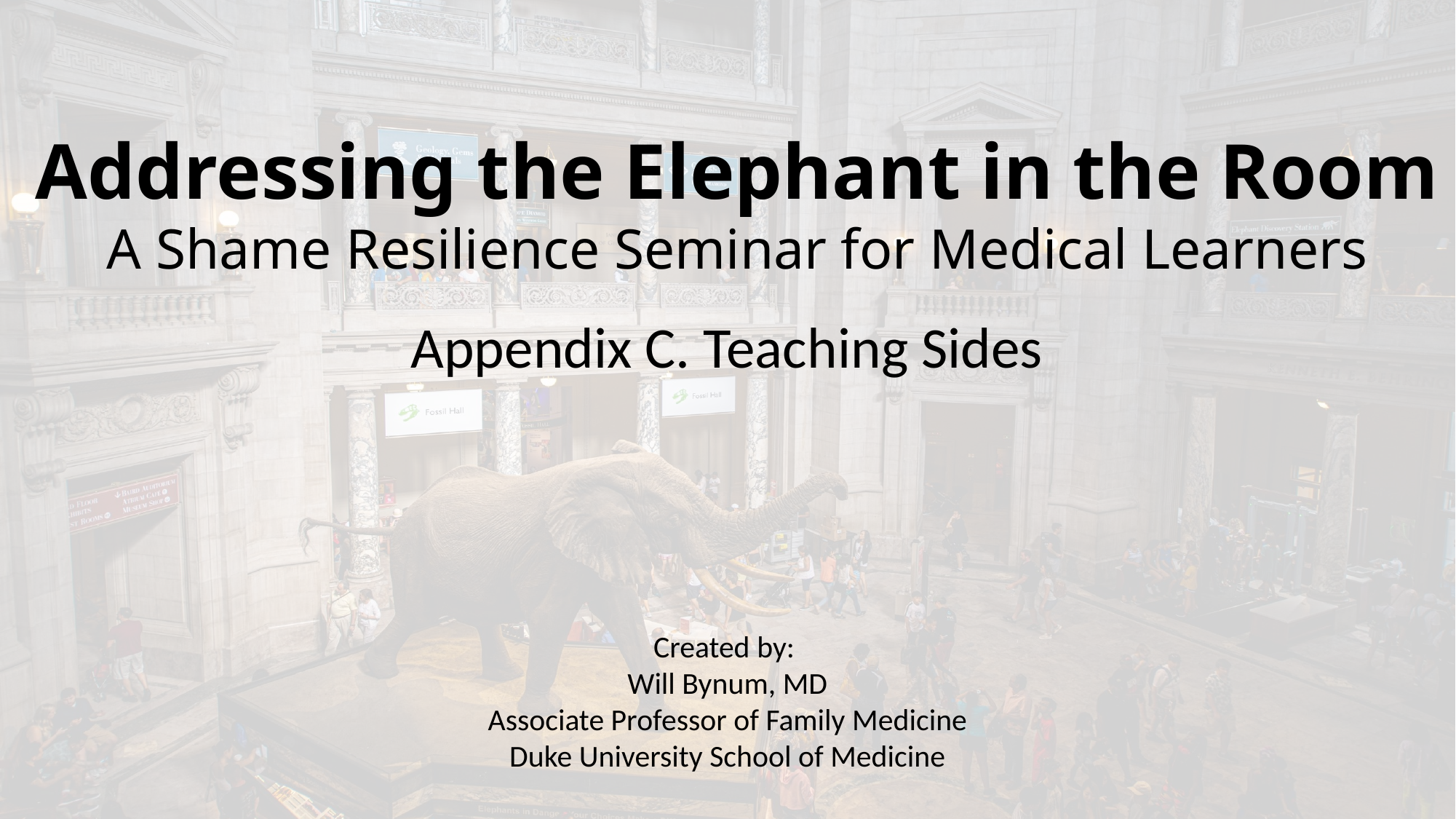

Addressing the Elephant in the Room
A Shame Resilience Seminar for Medical Learners
Appendix C. Teaching Sides
Created by:
Will Bynum, MD
Associate Professor of Family Medicine
Duke University School of Medicine

## Slide 2
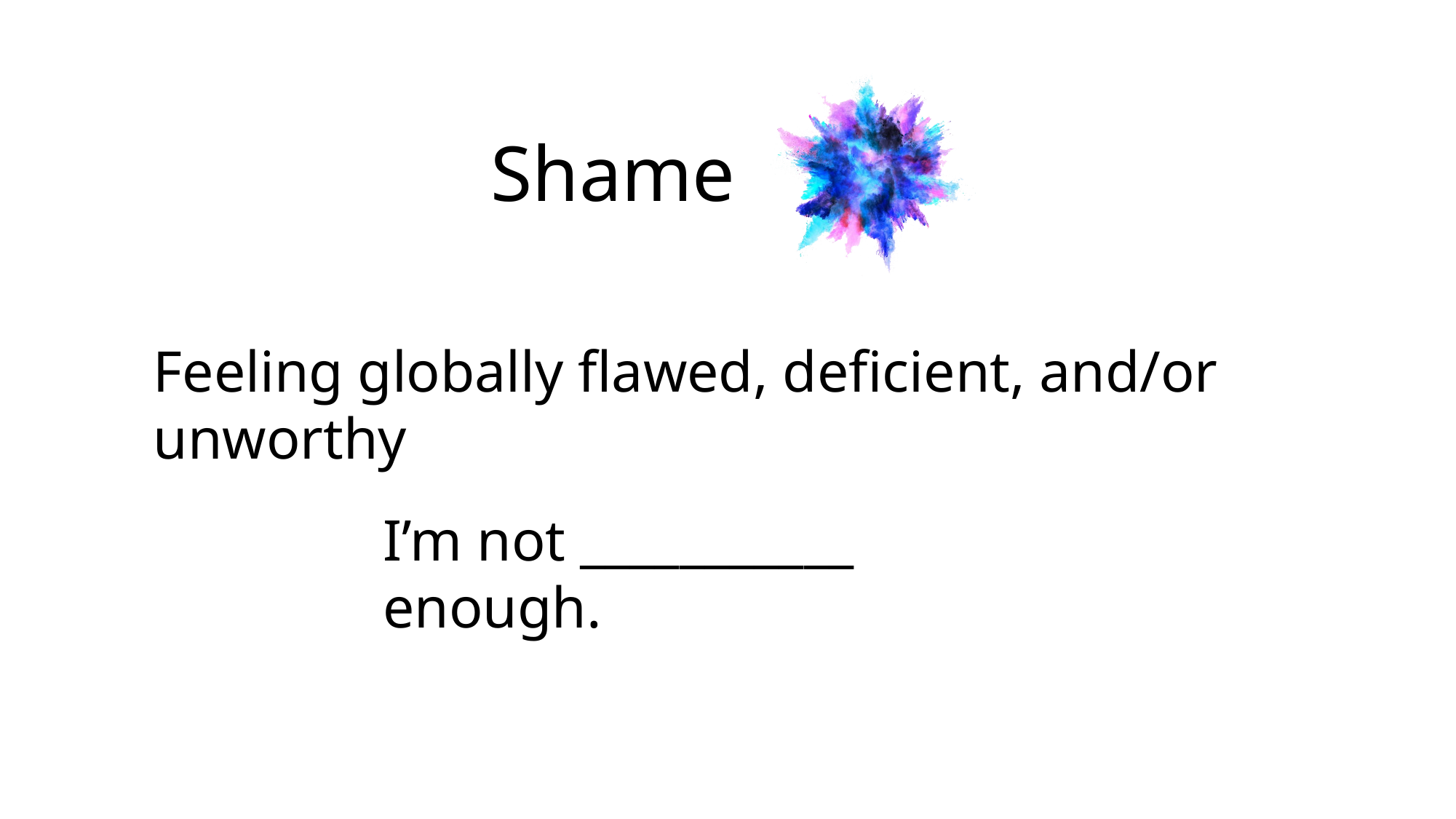

Shame
Feeling globally flawed, deficient, and/or unworthy
I’m not ___________ enough.

## Slide 3
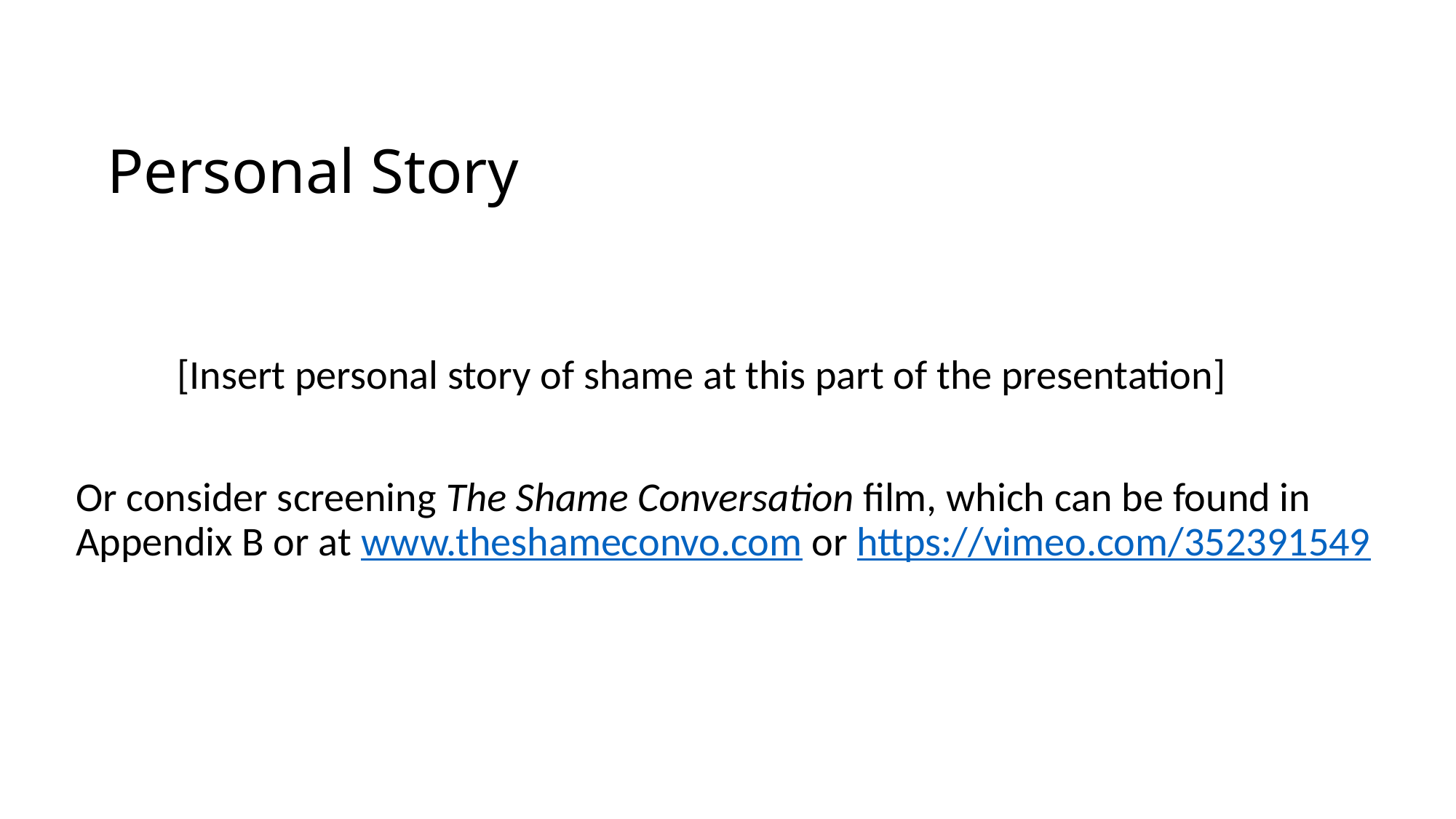

# Personal Story
[Insert personal story of shame at this part of the presentation]
Or consider screening The Shame Conversation film, which can be found in Appendix B or at www.theshameconvo.com or https://vimeo.com/352391549

## Slide 4
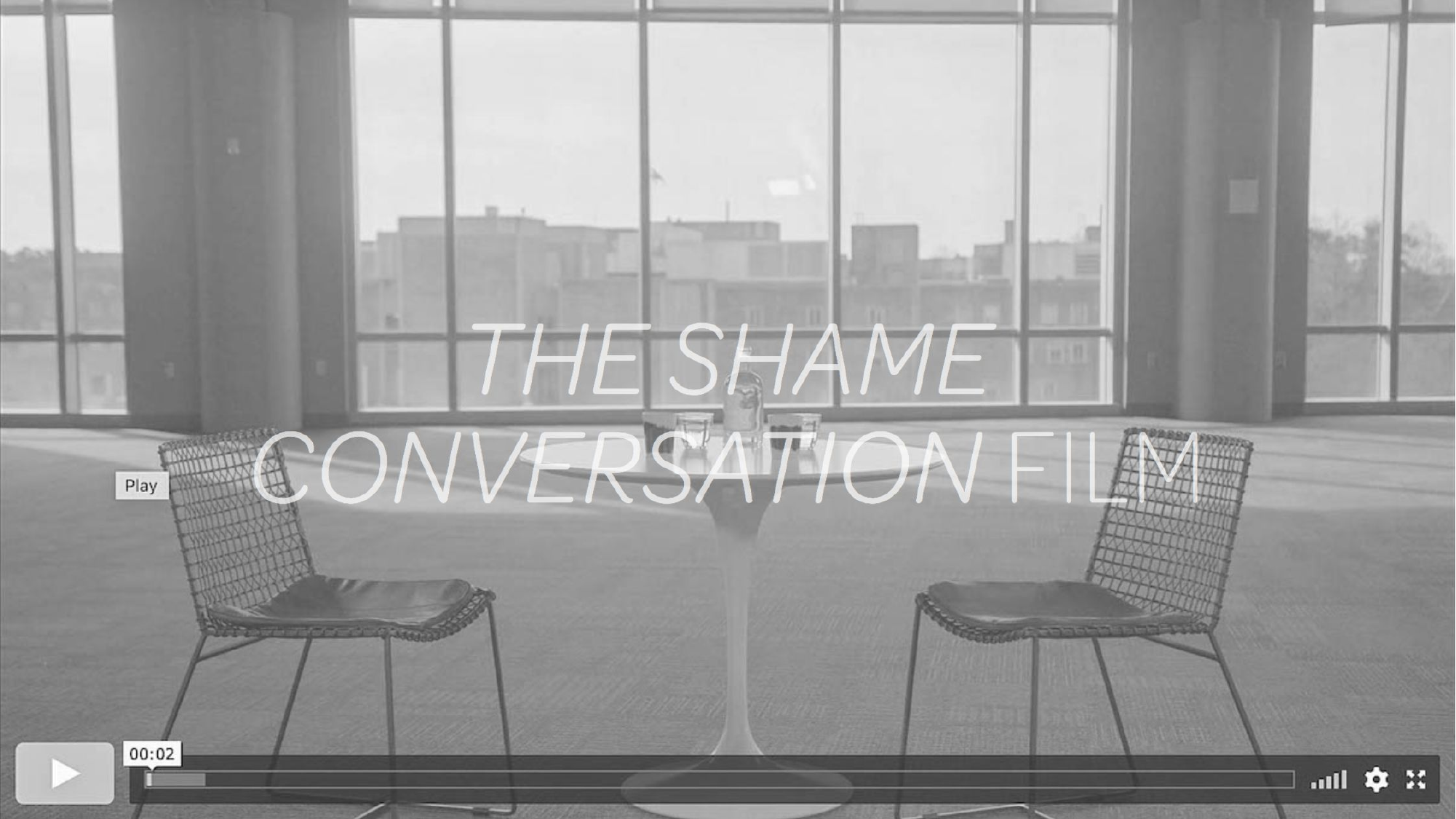

## Slide 5
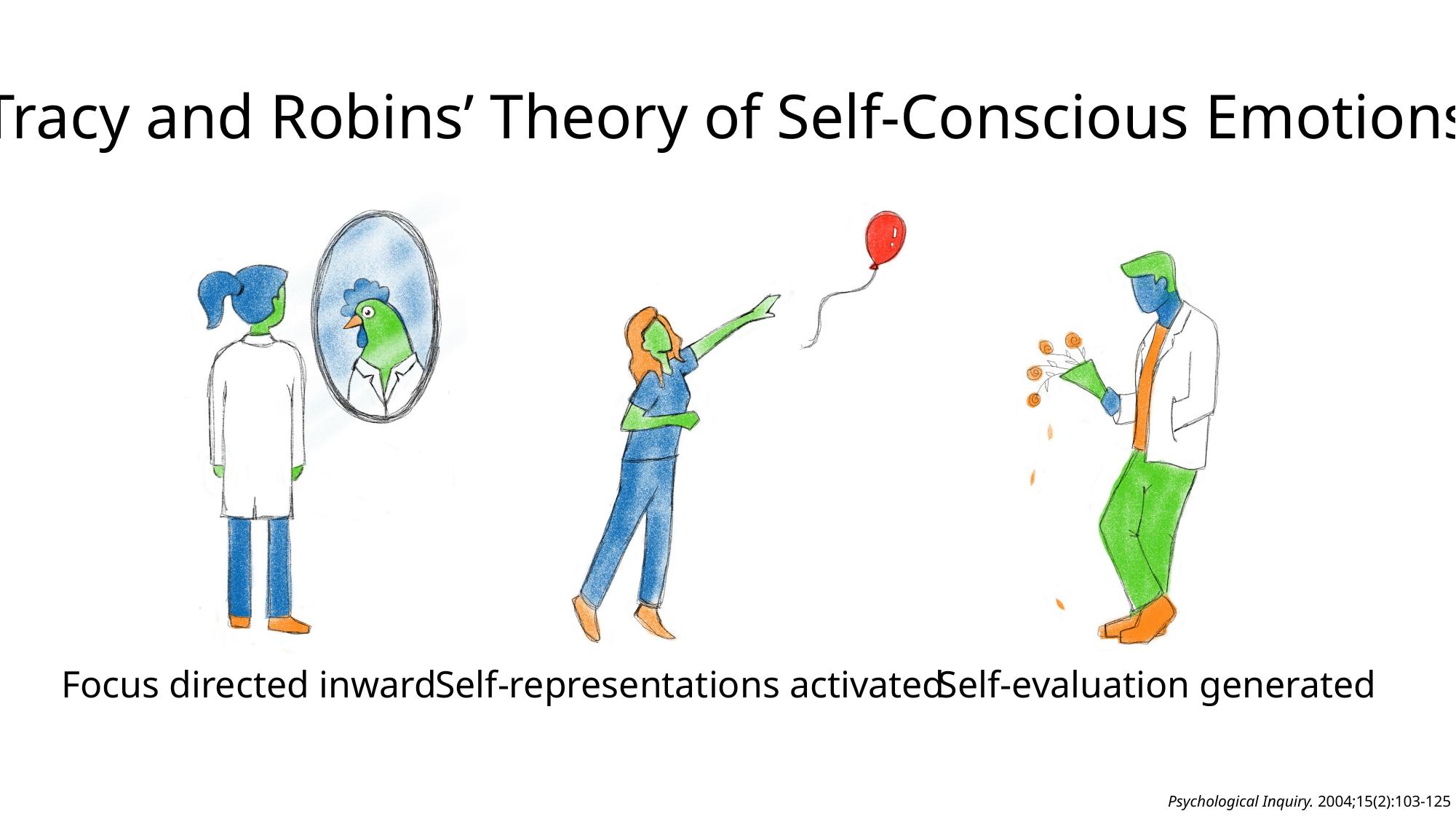

Tracy and Robins’ Theory of Self-Conscious Emotions
Focus directed inward
Self-representations activated
Self-evaluation generated
Psychological Inquiry. 2004;15(2):103-125

## Slide 6
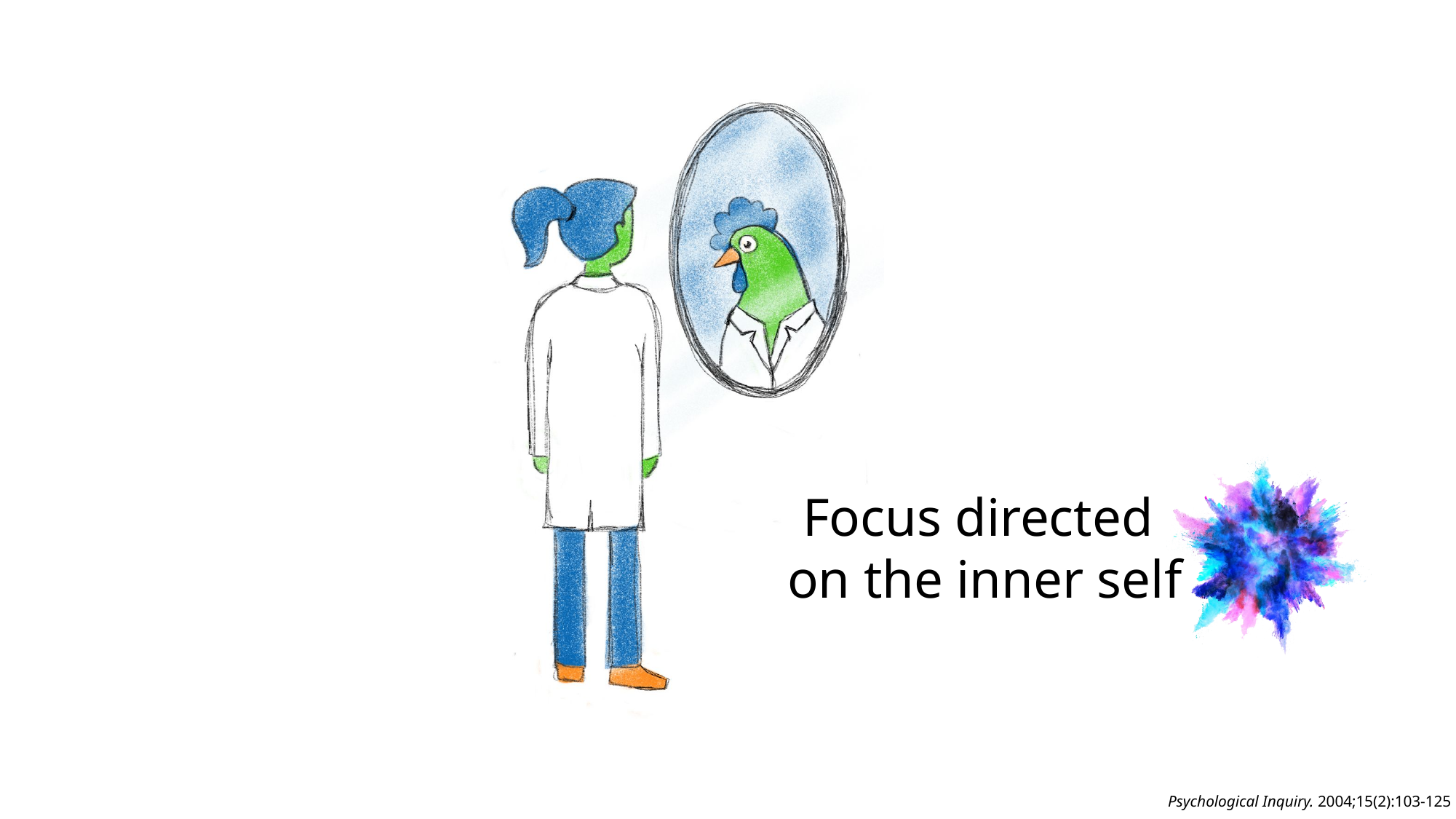

Focus directed
on the inner self
Psychological Inquiry. 2004;15(2):103-125

## Slide 7
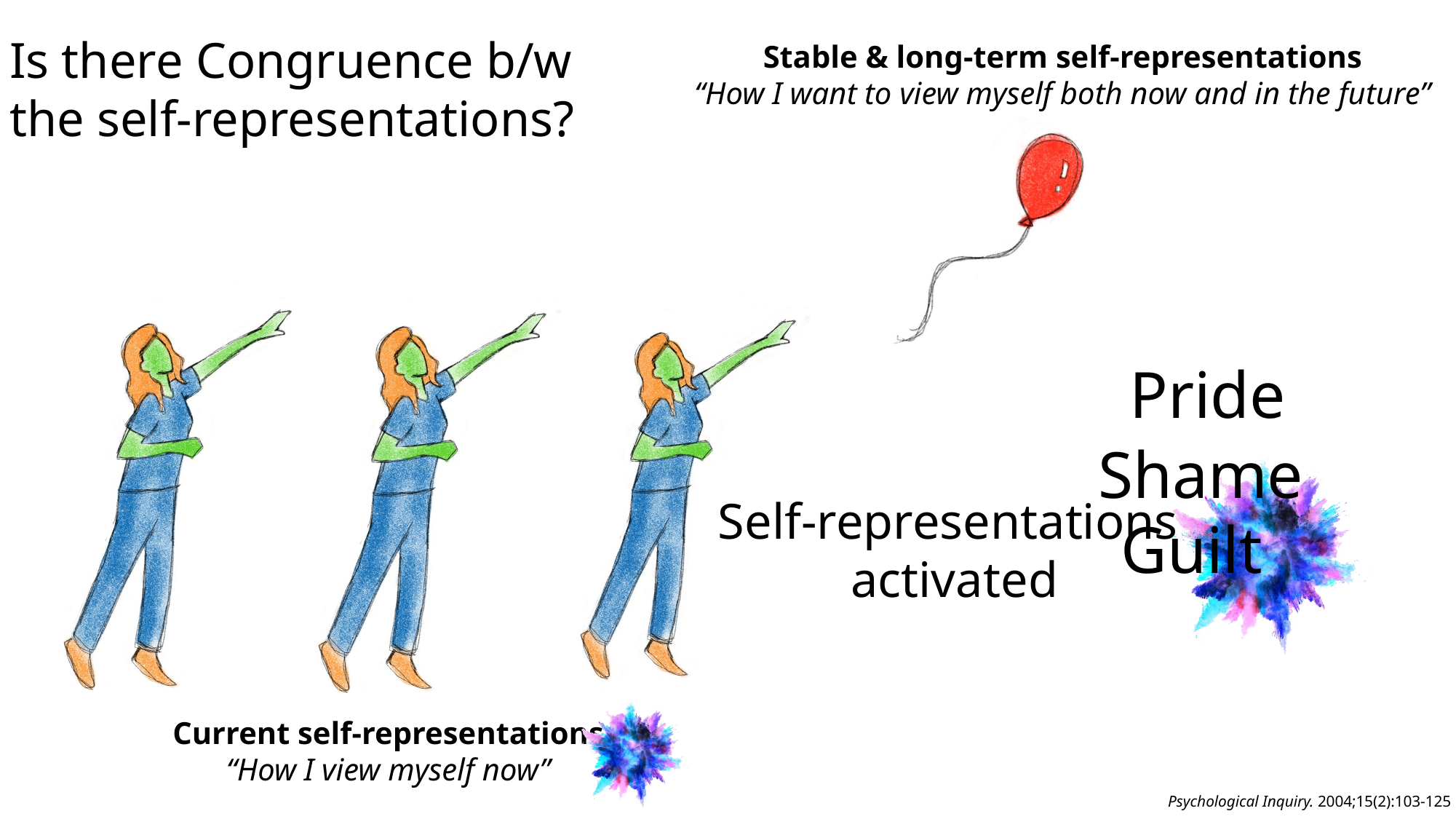

Is there Congruence b/w
the self-representations?
Stable & long-term self-representations
“How I want to view myself both now and in the future”
Pride
Shame
Self-representations
activated
Guilt
Current self-representations
“How I view myself now”
Psychological Inquiry. 2004;15(2):103-125

## Slide 8
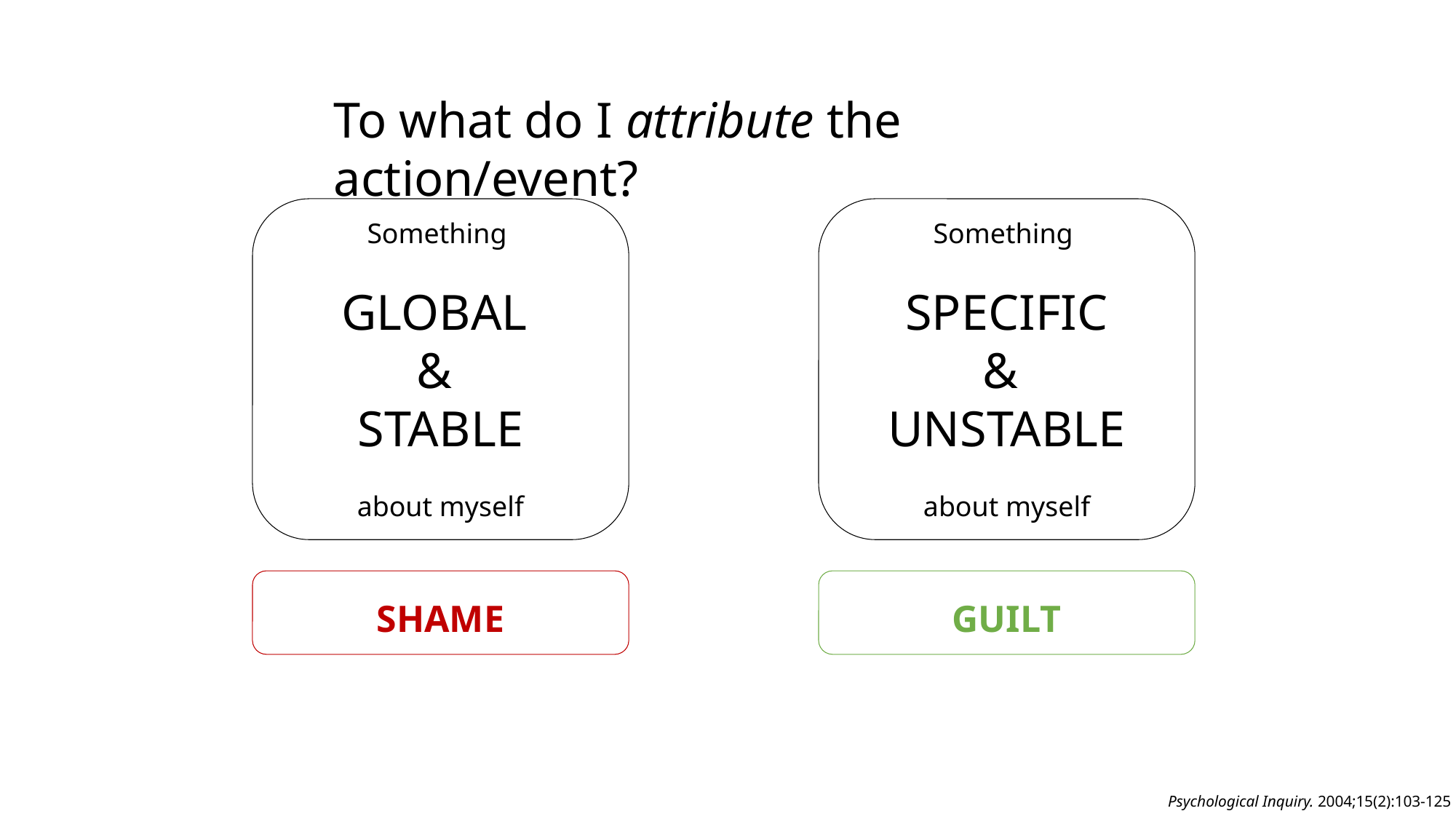

To what do I attribute the action/event?
Something
GLOBAL
&
STABLE
about myself
Something
SPECIFIC
&
UNSTABLE
about myself
SHAME
GUILT
Psychological Inquiry. 2004;15(2):103-125

## Slide 9
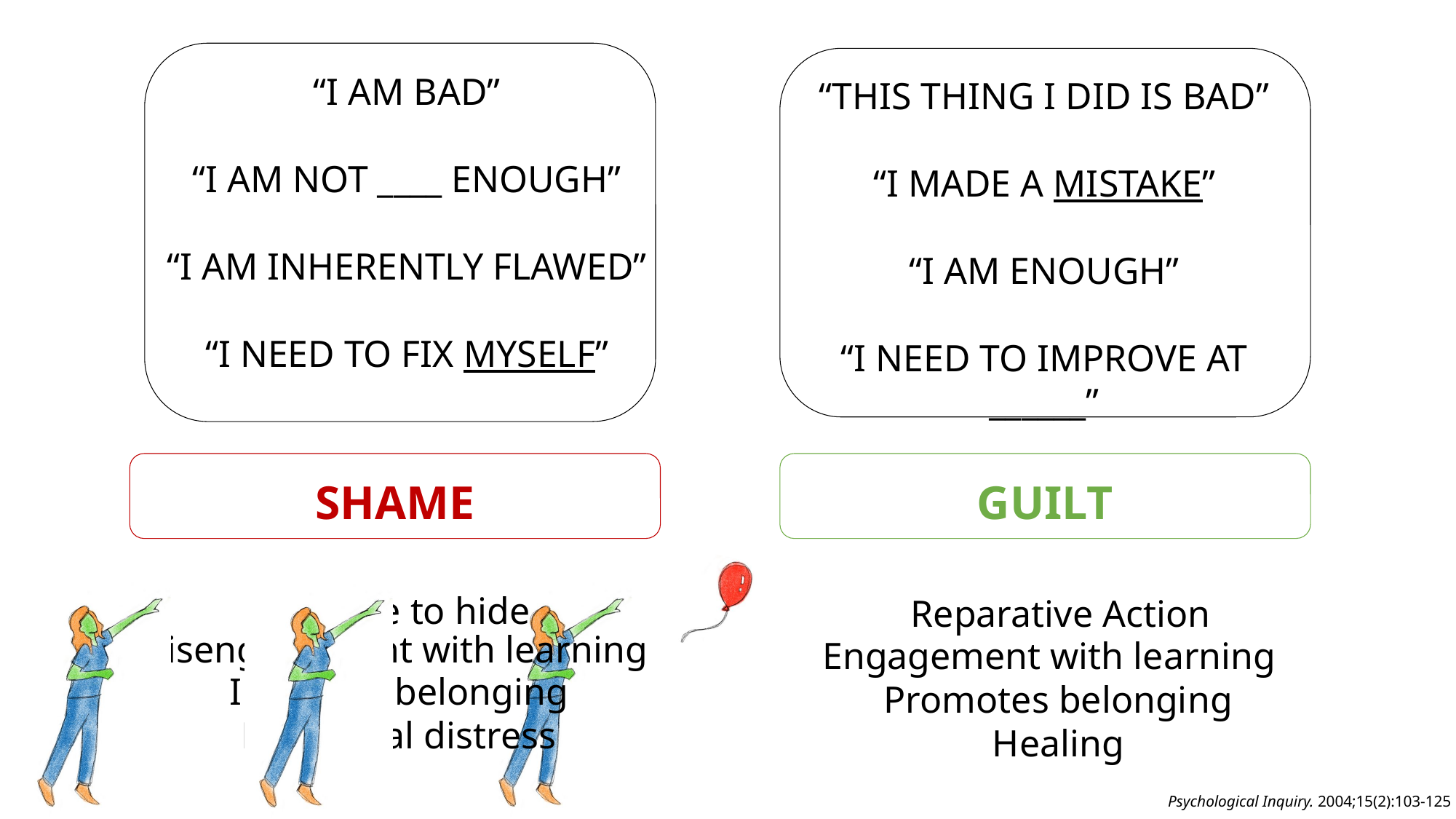

“I AM BAD”
“I AM NOT ____ ENOUGH”
“I AM INHERENTLY FLAWED”
“I NEED TO FIX MYSELF”
“THIS THING I DID IS BAD”
“I MADE A MISTAKE”
“I AM ENOUGH”
“I NEED TO IMPROVE AT ______”
SHAME
GUILT
Desire to hide
Reparative Action
Disengagement with learning
Engagement with learning
Impaired belonging
Emotional distress
Promotes belonging
Healing
Psychological Inquiry. 2004;15(2):103-125

## Slide 10
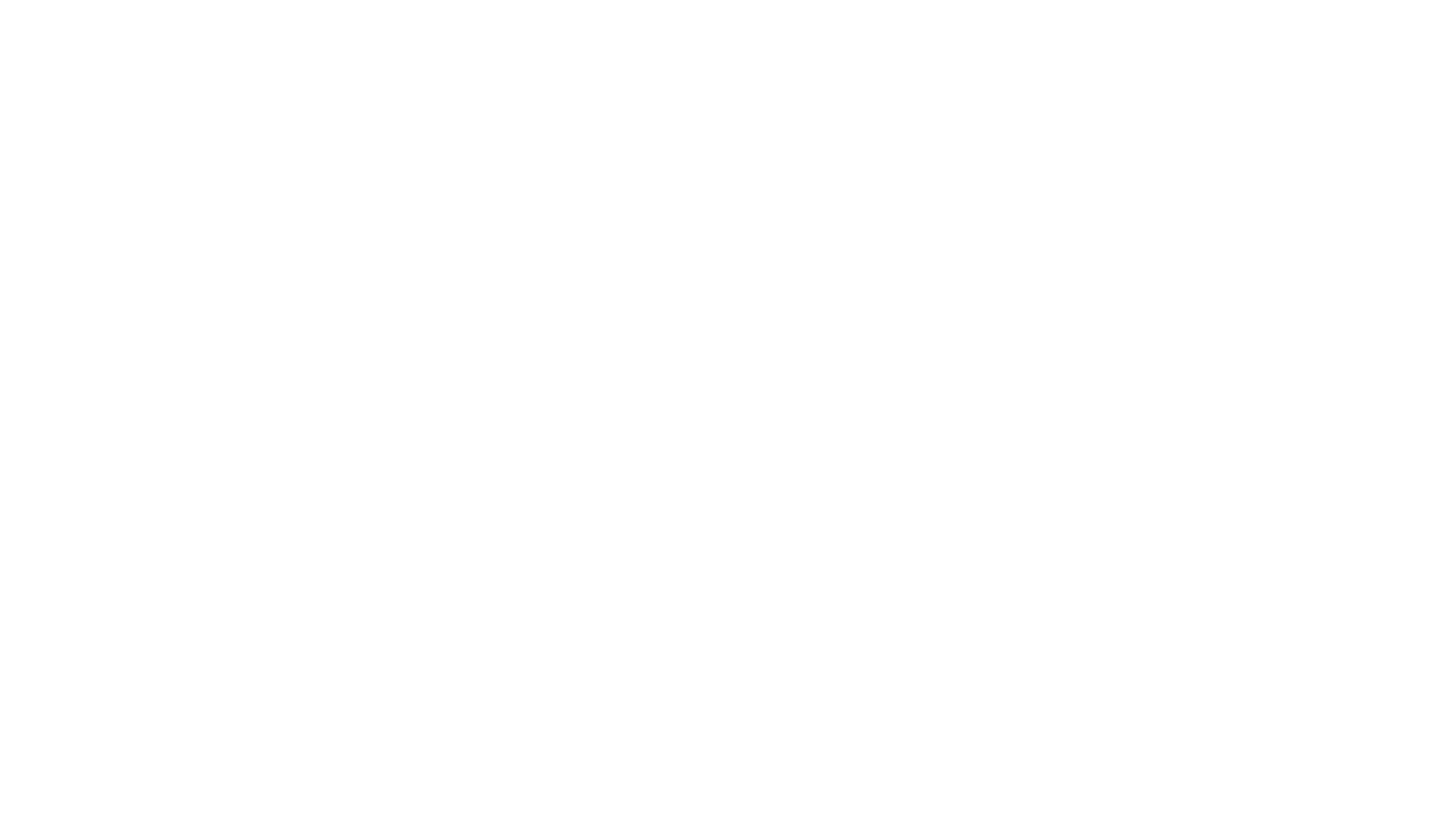

## Slide 11
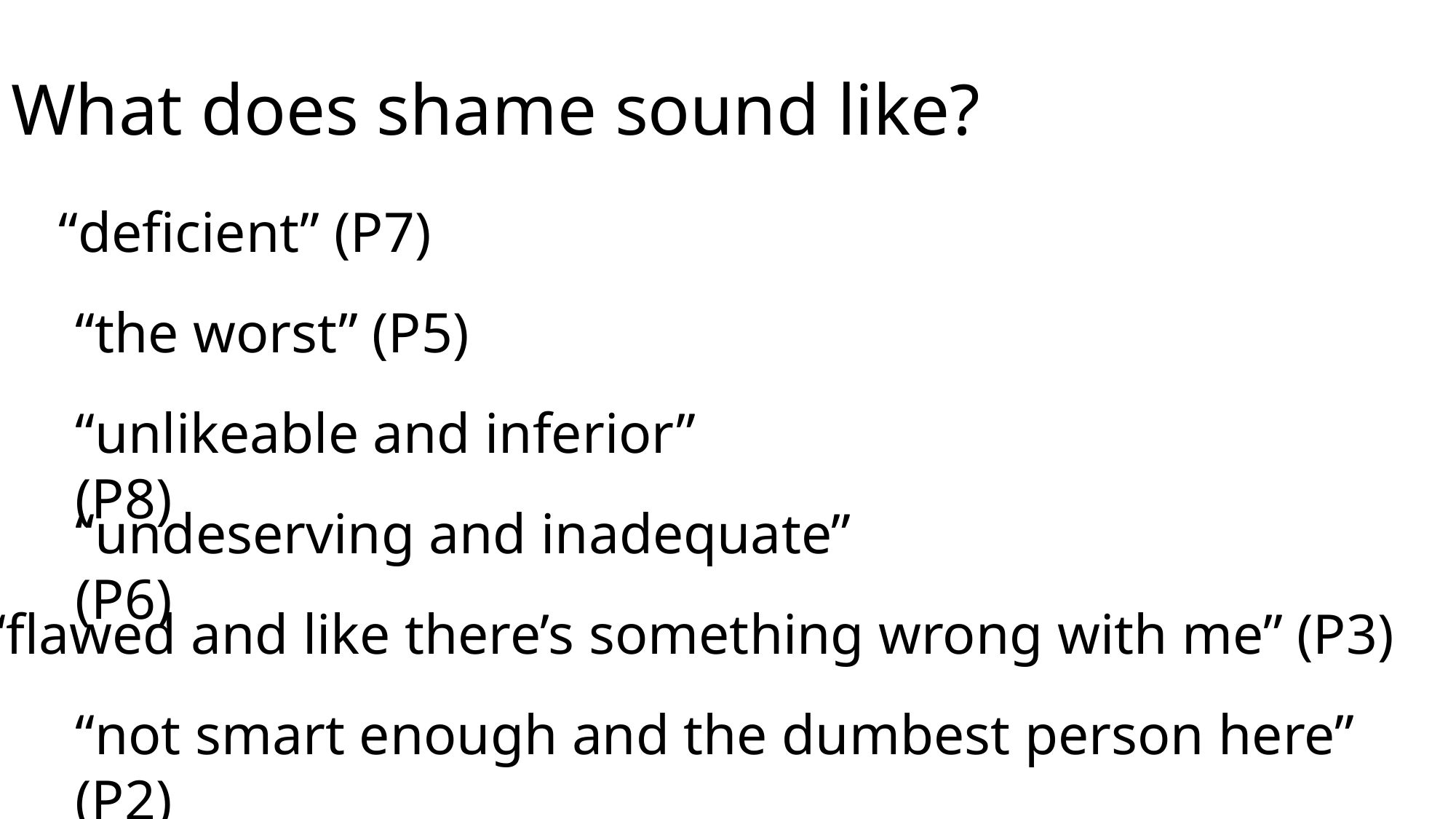

What does shame sound like?
“deficient” (P7)
“the worst” (P5)
“unlikeable and inferior” (P8)
“undeserving and inadequate” (P6)
“flawed and like there’s something wrong with me” (P3)
“not smart enough and the dumbest person here” (P2)

## Slide 12
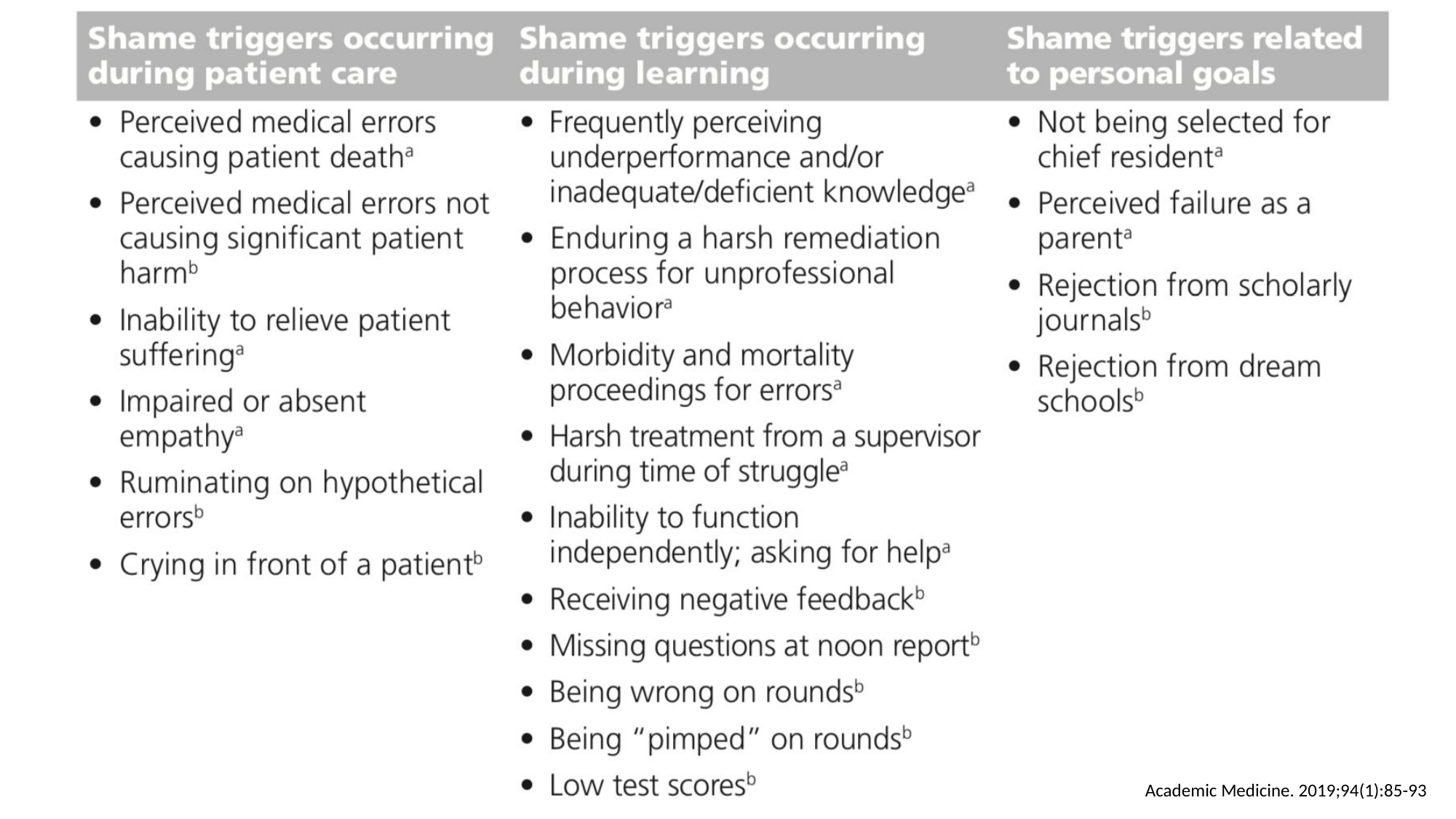

Academic Medicine. 2019;94(1):85-93

## Slide 13
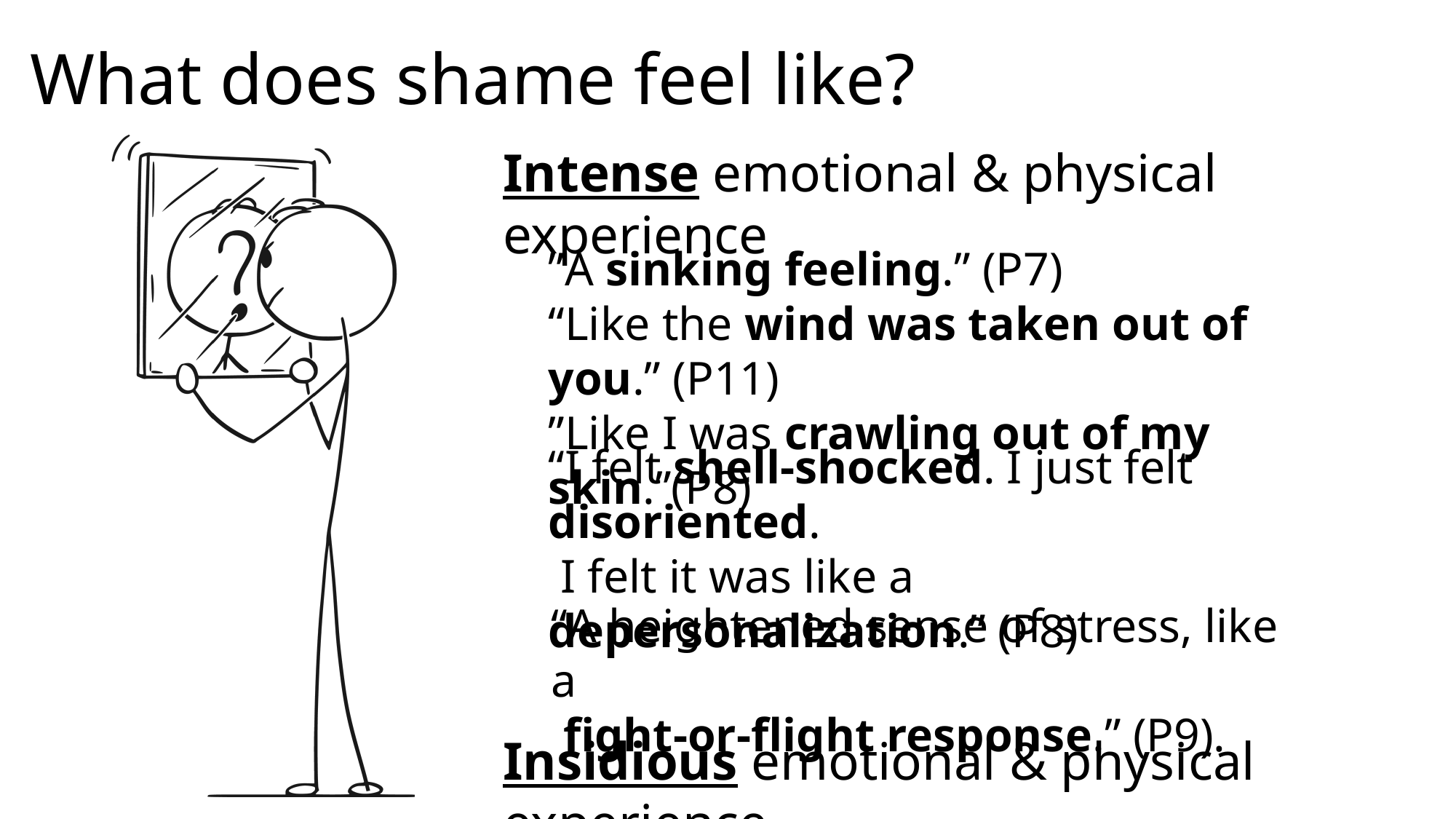

What does shame feel like?
Intense emotional & physical experience
”A sinking feeling.” (P7)
“Like the wind was taken out of you.” (P11)
”Like I was crawling out of my skin.”(P8)
“I felt shell-shocked. I just felt disoriented.
 I felt it was like a depersonalization.” (P8)
“A heightened sense of stress, like a
 fight-or-flight response.” (P9).
Insidious emotional & physical experience

## Slide 14
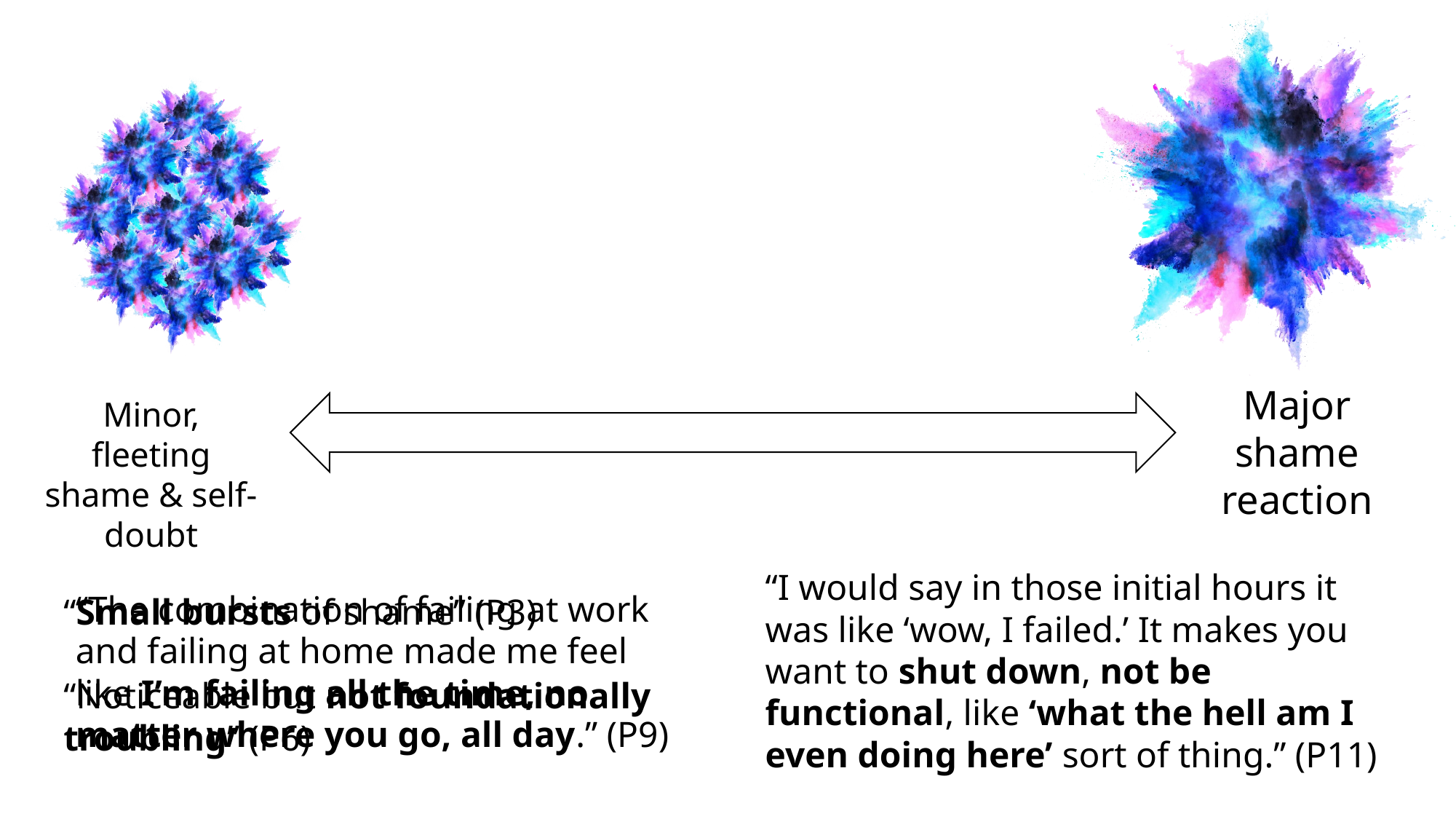

Major shame reaction
Minor, fleeting shame & self-doubt
“I would say in those initial hours it was like ‘wow, I failed.’ It makes you want to shut down, not be functional, like ‘what the hell am I even doing here’ sort of thing.” (P11)
“The combination of failing at work and failing at home made me feel like I’m failing all the time, no matter where you go, all day.” (P9)
“Small bursts of shame” (P3)
“Noticeable but not foundationally troubling” (P6)

## Slide 15
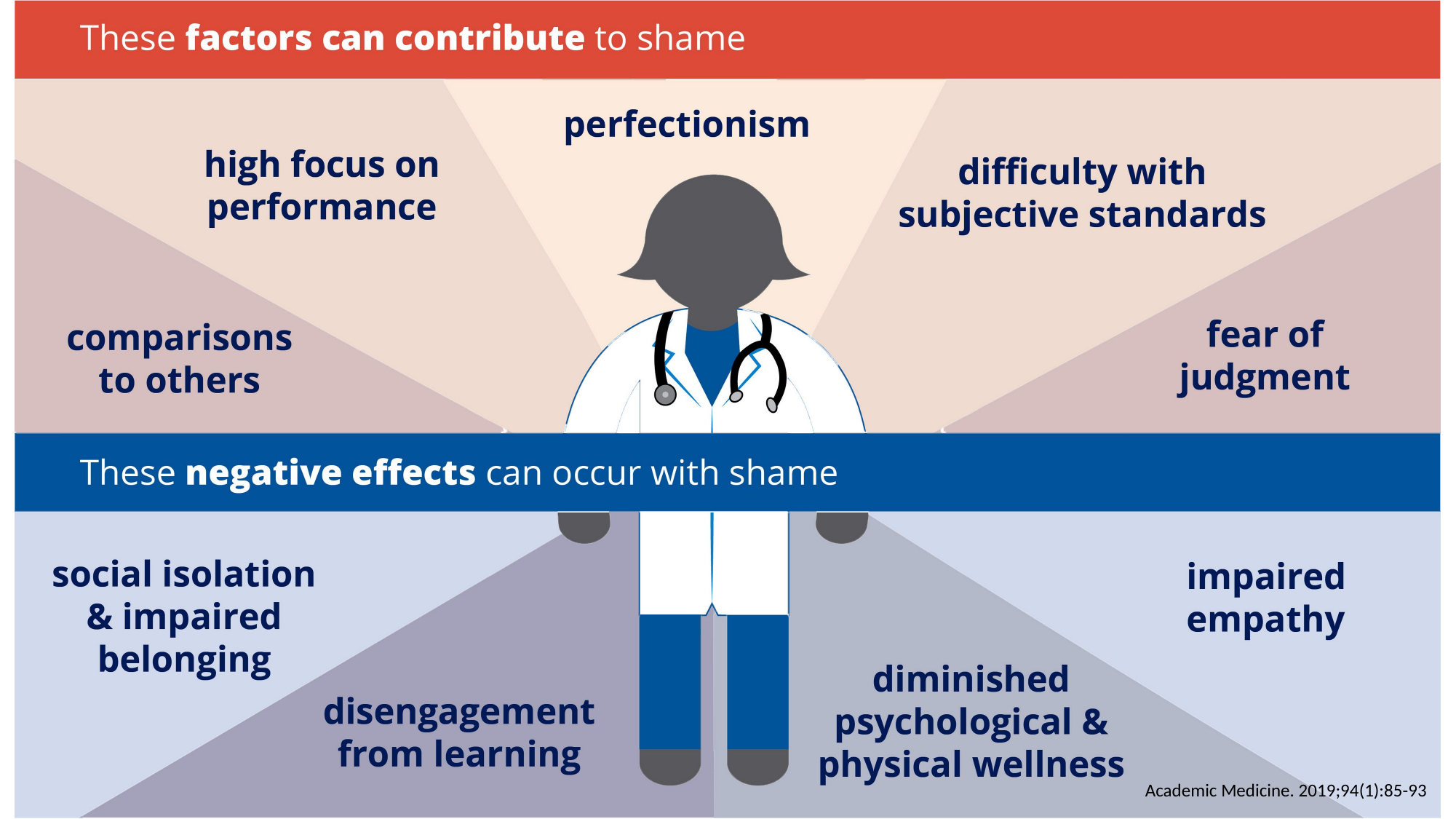

Academic Medicine. 2019;94(1):85-93

## Slide 16
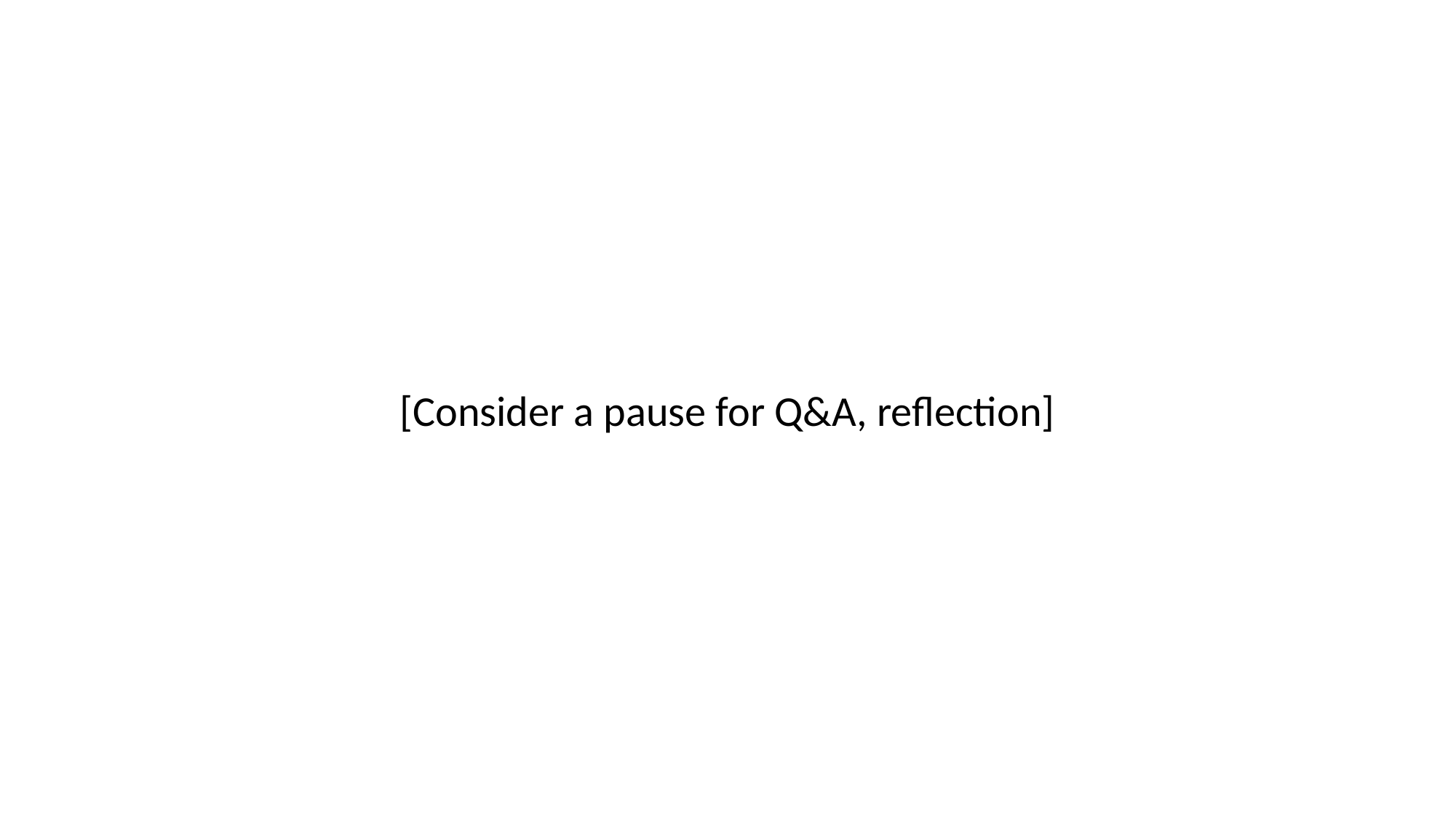

[Consider a pause for Q&A, reflection]

## Slide 17
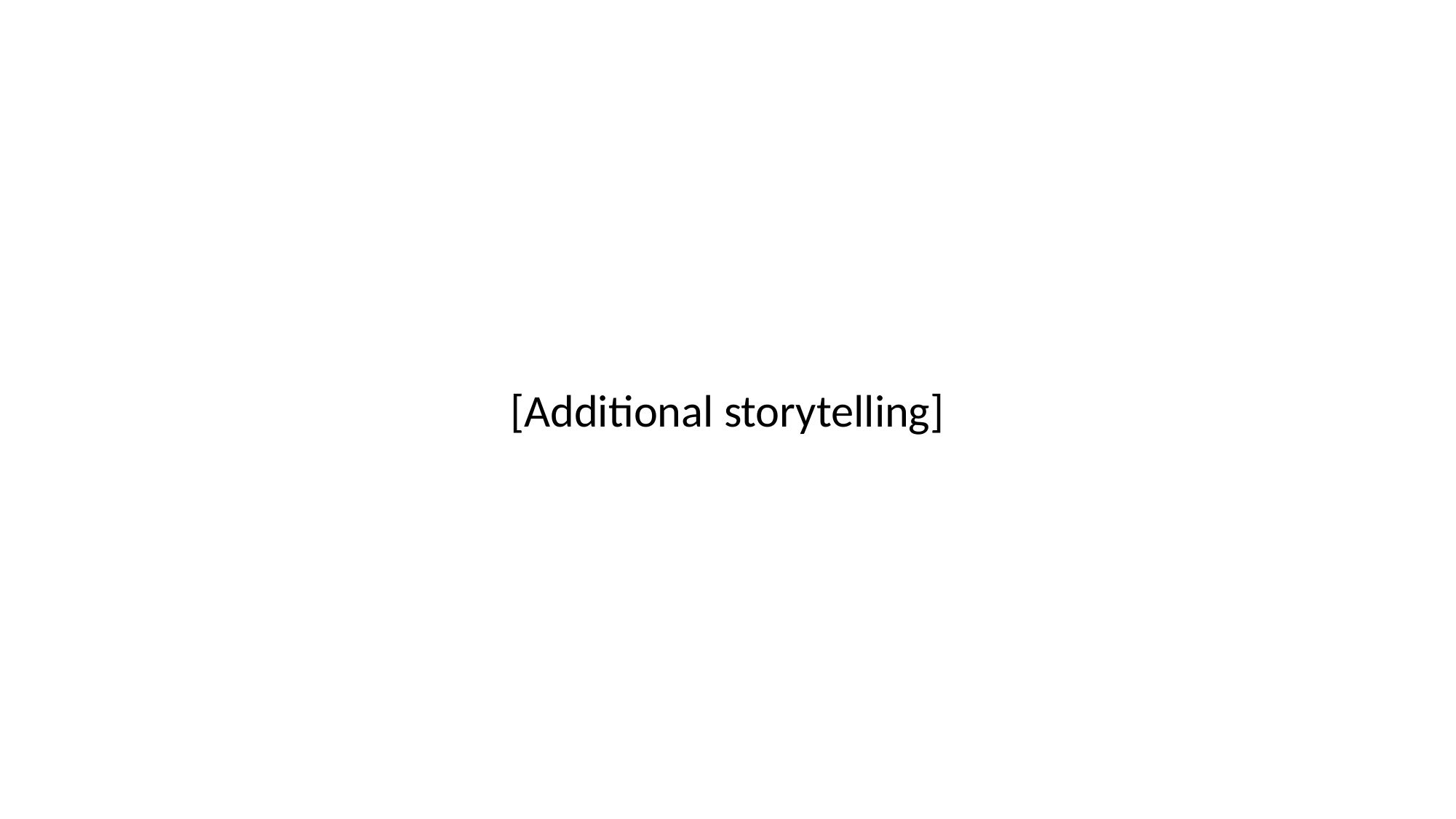

[Additional storytelling]

## Slide 18
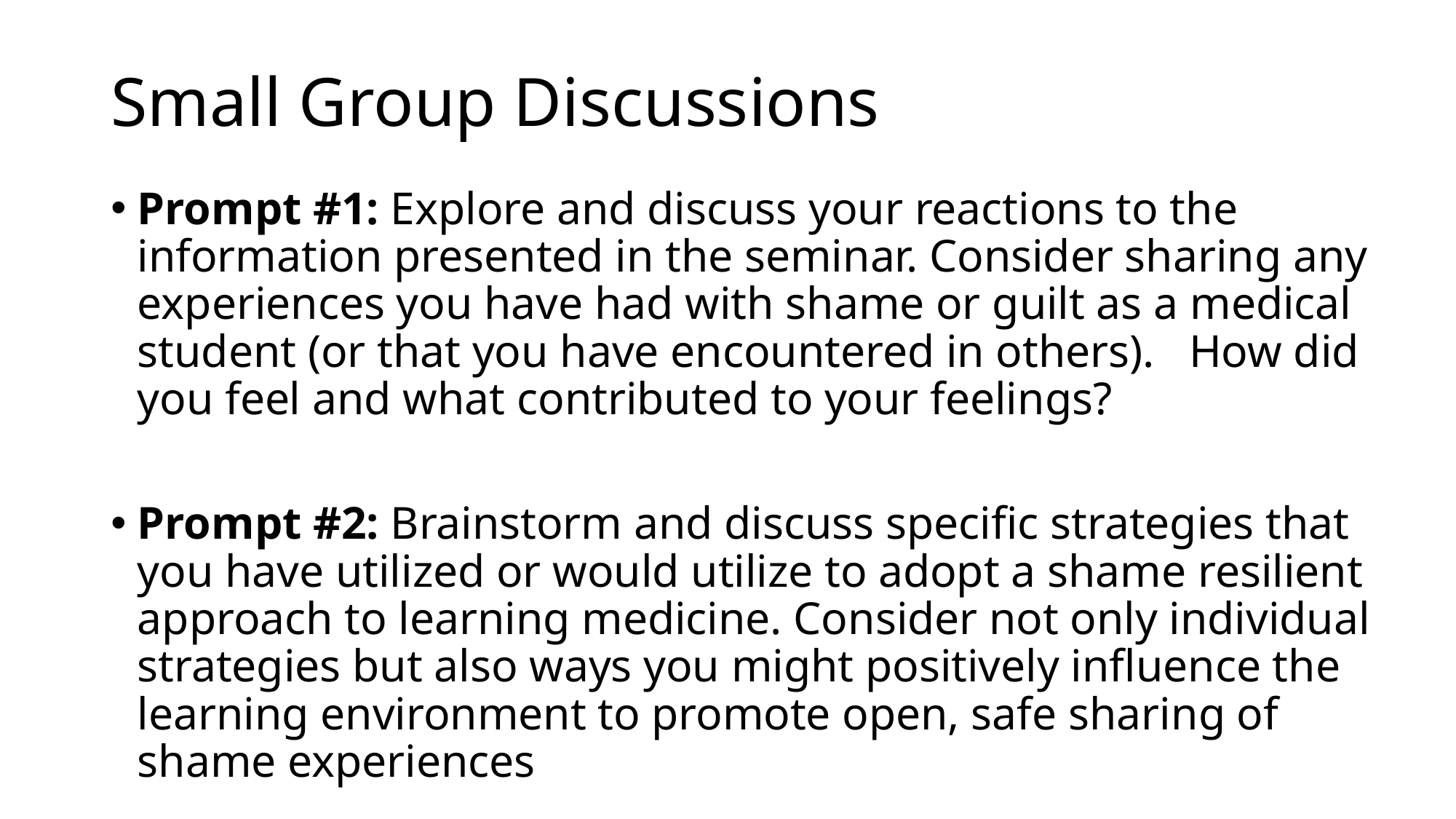

# Small Group Discussions
Prompt #1: Explore and discuss your reactions to the information presented in the seminar. Consider sharing any experiences you have had with shame or guilt as a medical student (or that you have encountered in others). How did you feel and what contributed to your feelings?
Prompt #2: Brainstorm and discuss specific strategies that you have utilized or would utilize to adopt a shame resilient approach to learning medicine. Consider not only individual strategies but also ways you might positively influence the learning environment to promote open, safe sharing of shame experiences

## Slide 19
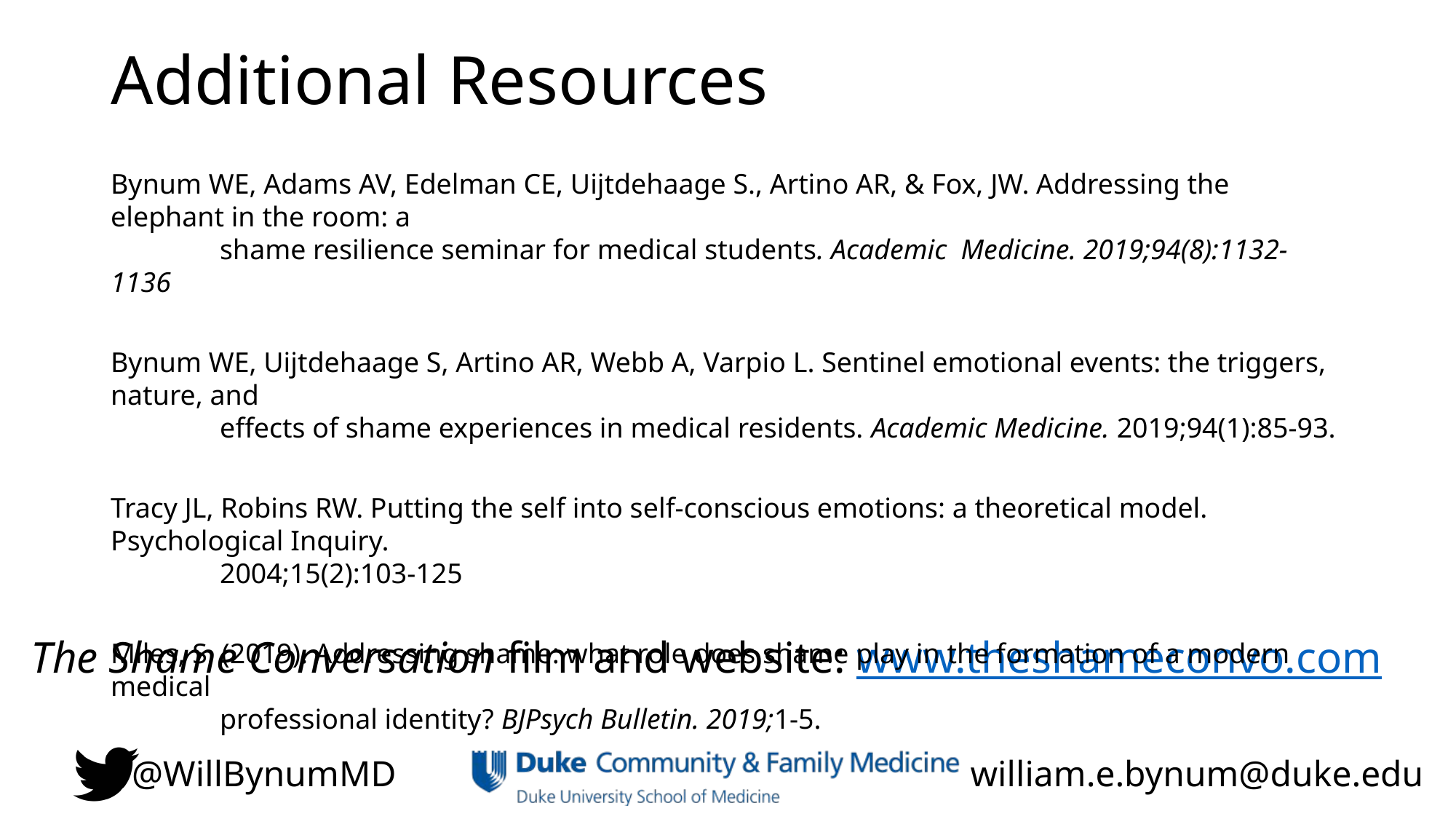

# Additional Resources
Bynum WE, Adams AV, Edelman CE, Uijtdehaage S., Artino AR, & Fox, JW. Addressing the elephant in the room: a
	shame resilience seminar for medical students. Academic Medicine. 2019;94(8):1132-1136
Bynum WE, Uijtdehaage S, Artino AR, Webb A, Varpio L. Sentinel emotional events: the triggers, nature, and
	effects of shame experiences in medical residents. Academic Medicine. 2019;94(1):85-93.
Tracy JL, Robins RW. Putting the self into self-conscious emotions: a theoretical model. Psychological Inquiry.
	2004;15(2):103-125
Miles, S. (2019). Addressing shame: what role does shame play in the formation of a modern medical
	professional identity? BJPsych Bulletin. 2019;1-5.
The Shame Conversation film and website: www.theshameconvo.com
@WillBynumMD
william.e.bynum@duke.edu
