# Supplementary material for: The Psychology of Shame: A Resilience Seminar for Medical Students
Source: MedEdPORTAL. 2020 Dec 24;16:11052. doi: 10.15766/mep_2374-8265.11052 (PMC7780736; doi:10.15766/mep_2374-8265.11052)
Supplement: Supplementary file 1 — Small Group Facilitator Guide.docxThe Shame Conversation Film.mp4Didactic Slides.pptxSmall Group Discussion Prompts.docxWorkshop Evaluations.docx [file mep_2374-8265.11052-s001.zip › D. Small Group Discussion Prompts.docx]

**Addressing the elephant in the room: a shame resilience seminar for medical students**

**Appendix D. Discussion Prompts for Small Group Facilitators**

**Prompt #1:** Explore and discuss your reactions to the information presented in the seminar. Consider sharing any experiences you have had with shame or guilt as a medical student (or that you have encountered in others). How did you feel and what contributed to your feelings?

**Prompt #2:** Brainstorm and discuss specific strategies that you have utilized or would utilize to adopt a shame resilient approach to learning medicine. Consider not only individual strategies but also ways you might positively influence the learning environment to promote open, safe sharing of shame experiences
