# Supplementary material for: The Psychology of Shame: A Resilience Seminar for Medical Students
Source: MedEdPORTAL. 2020 Dec 24;16:11052. doi: 10.15766/mep_2374-8265.11052 (PMC7780736; doi:10.15766/mep_2374-8265.11052)

**Addressing the Elephant in the Room**

A Shame Resilience Seminar for Medical Students

**
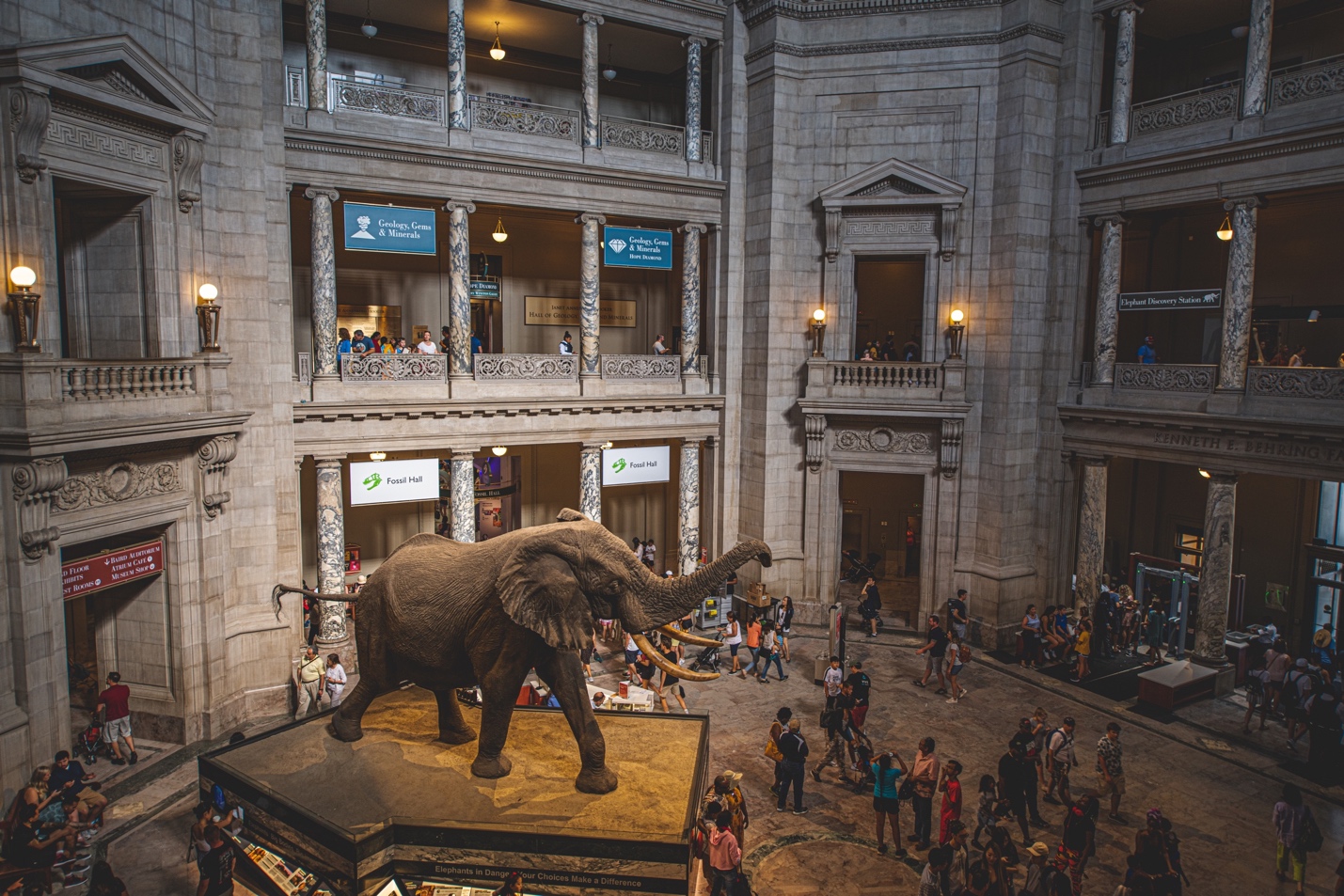
**

**Appendix E – Student & Faculty Surveys**

Prepared by Will Bynum, MD

Associate Professor of Family Medicine

Duke University School of Medicine

william.e.bynum@duke.edu

Image by mana5280, retrieved from [www.unsplash.com](http://www.unsplash.com) on Nov 10, 2019.

Creative Commons license associated: <https://unsplash.com/license>

**Student Survey**


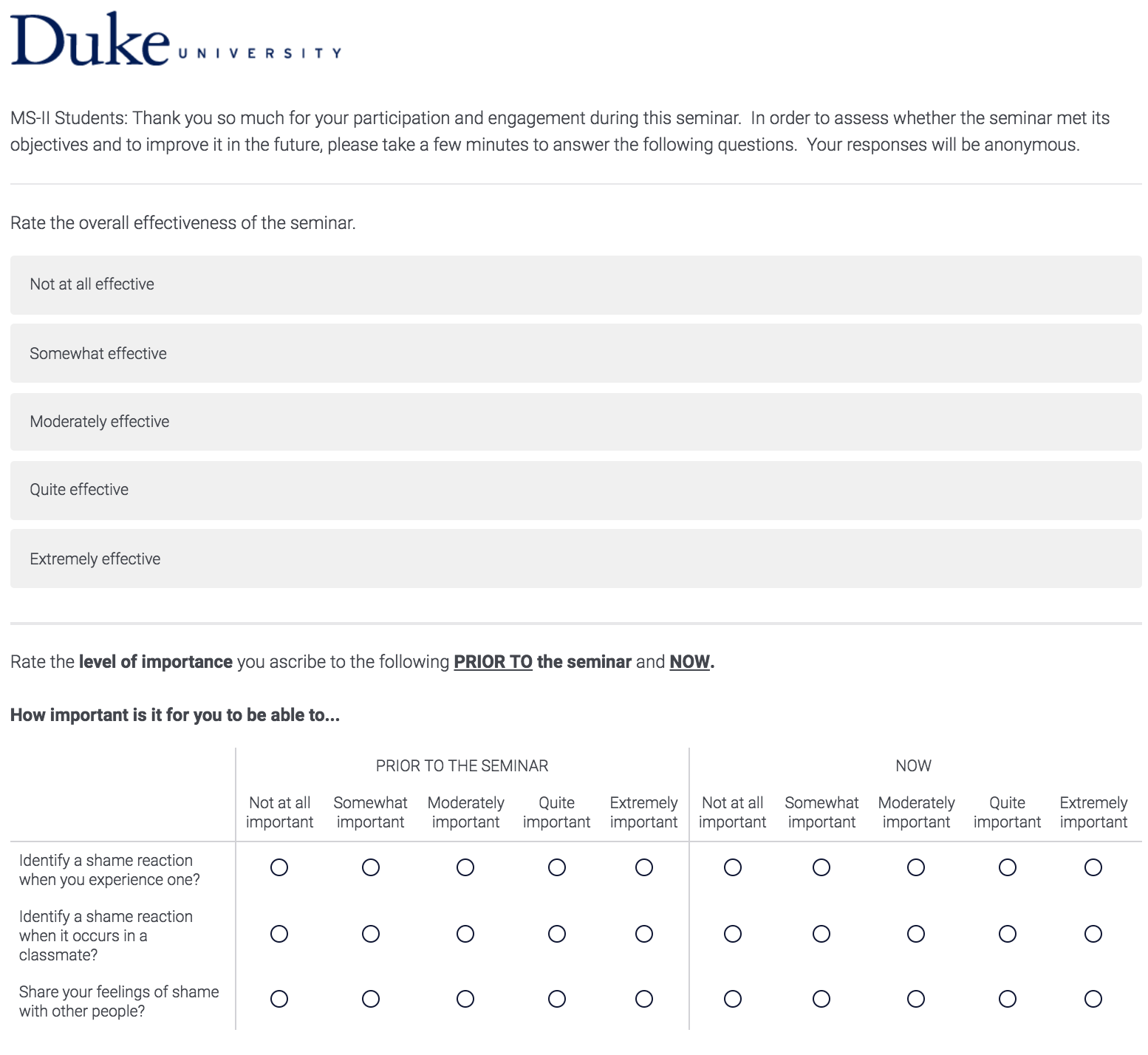


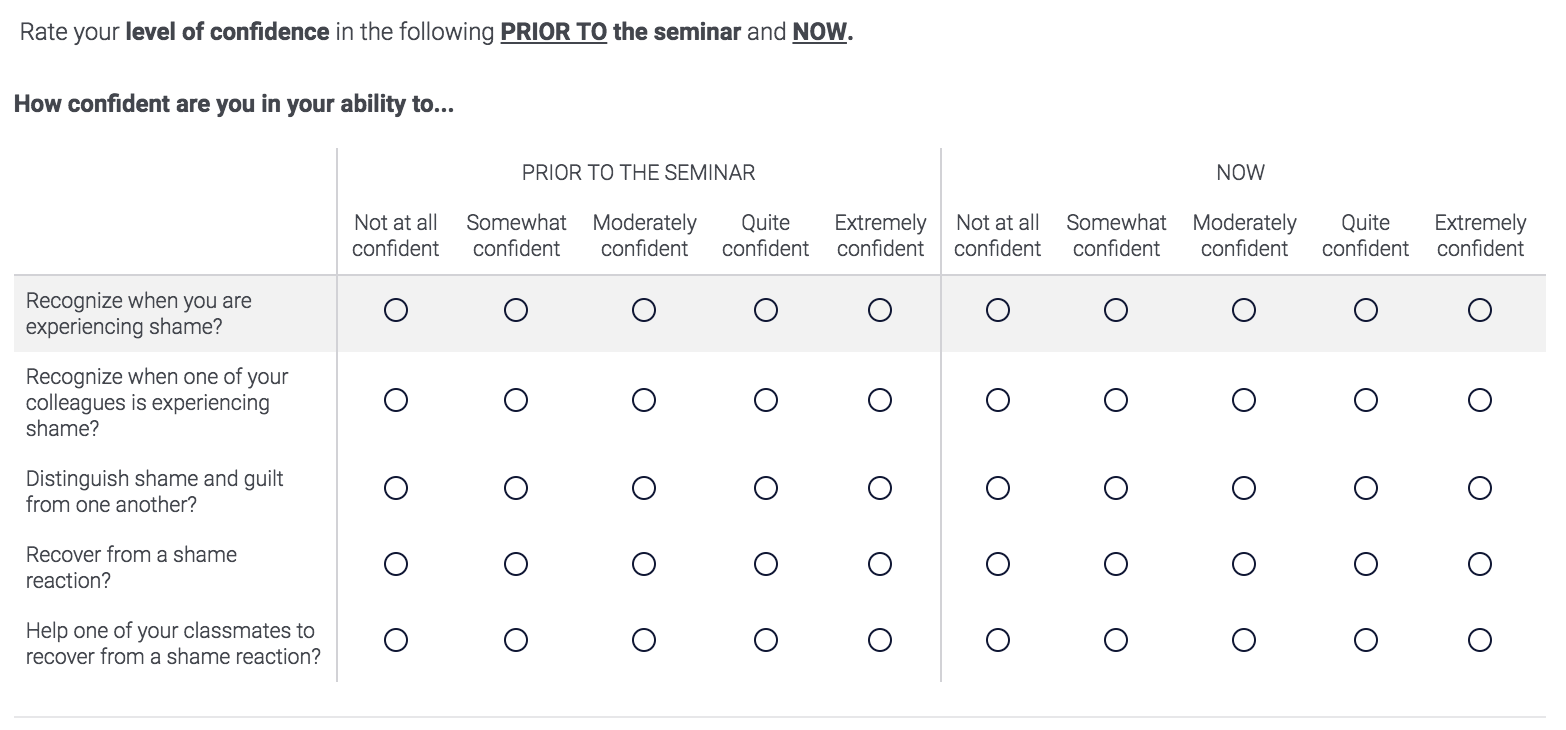


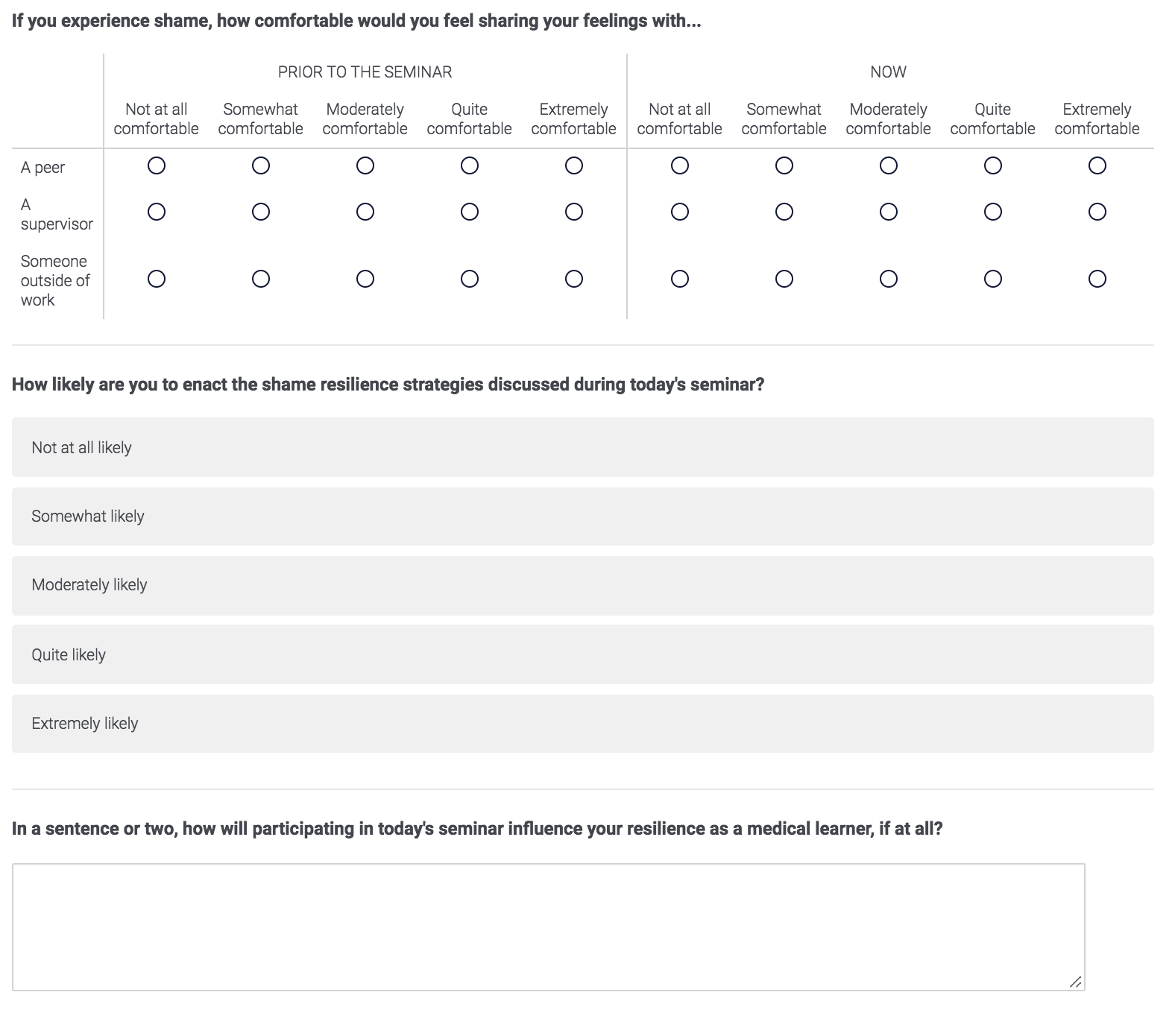


**
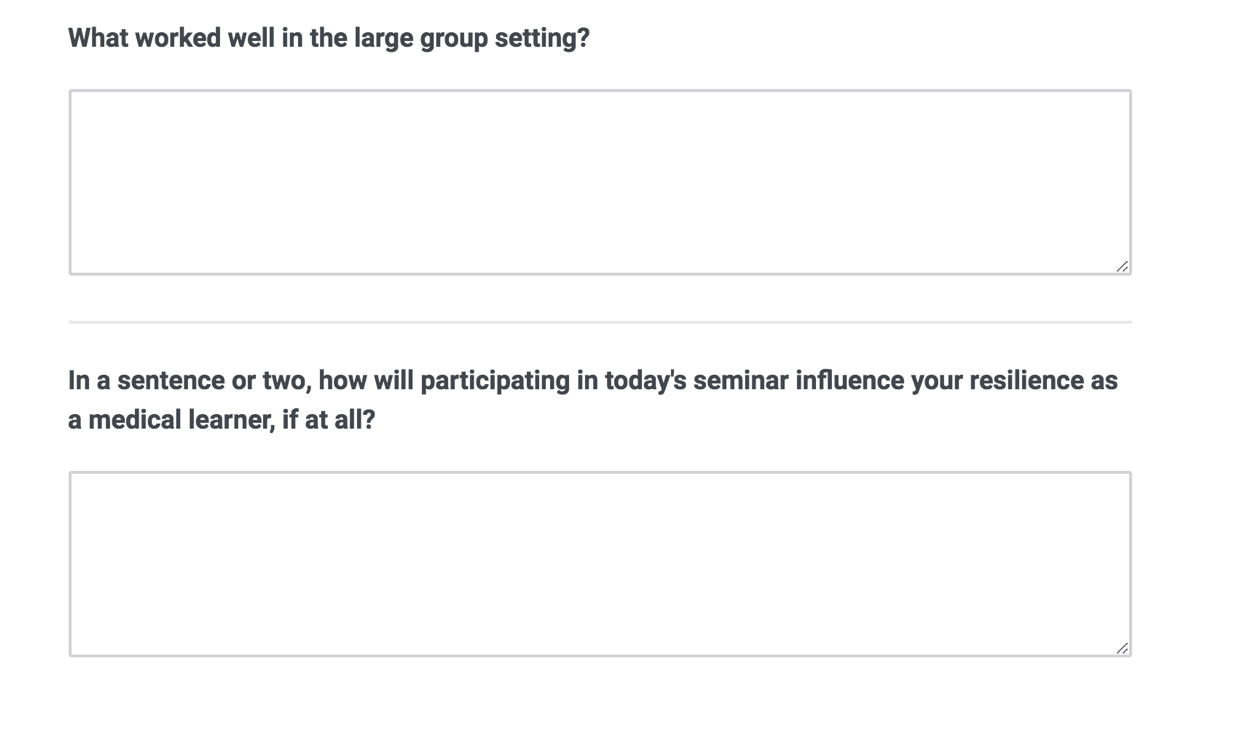
**

**Faculty Survey**


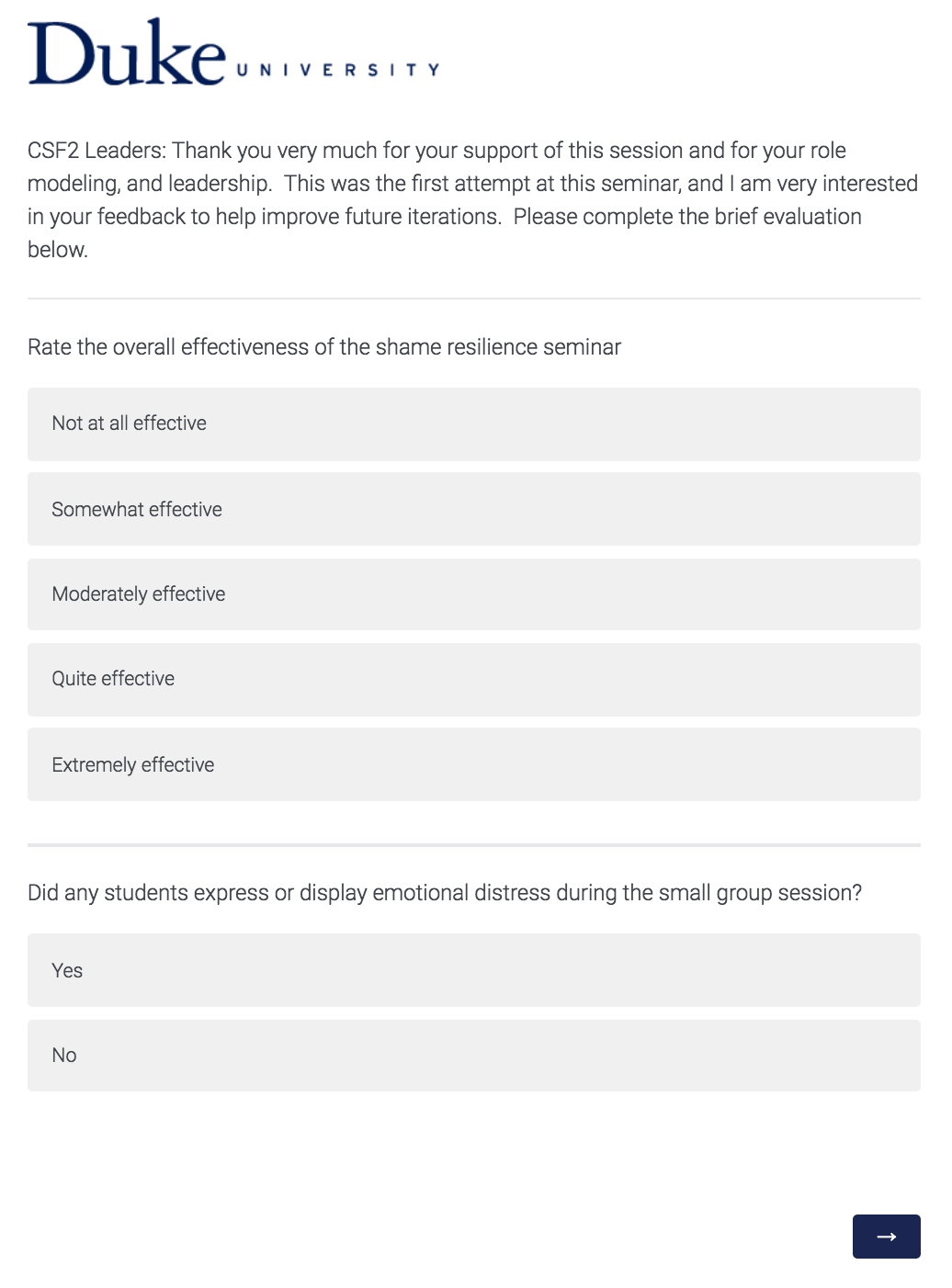


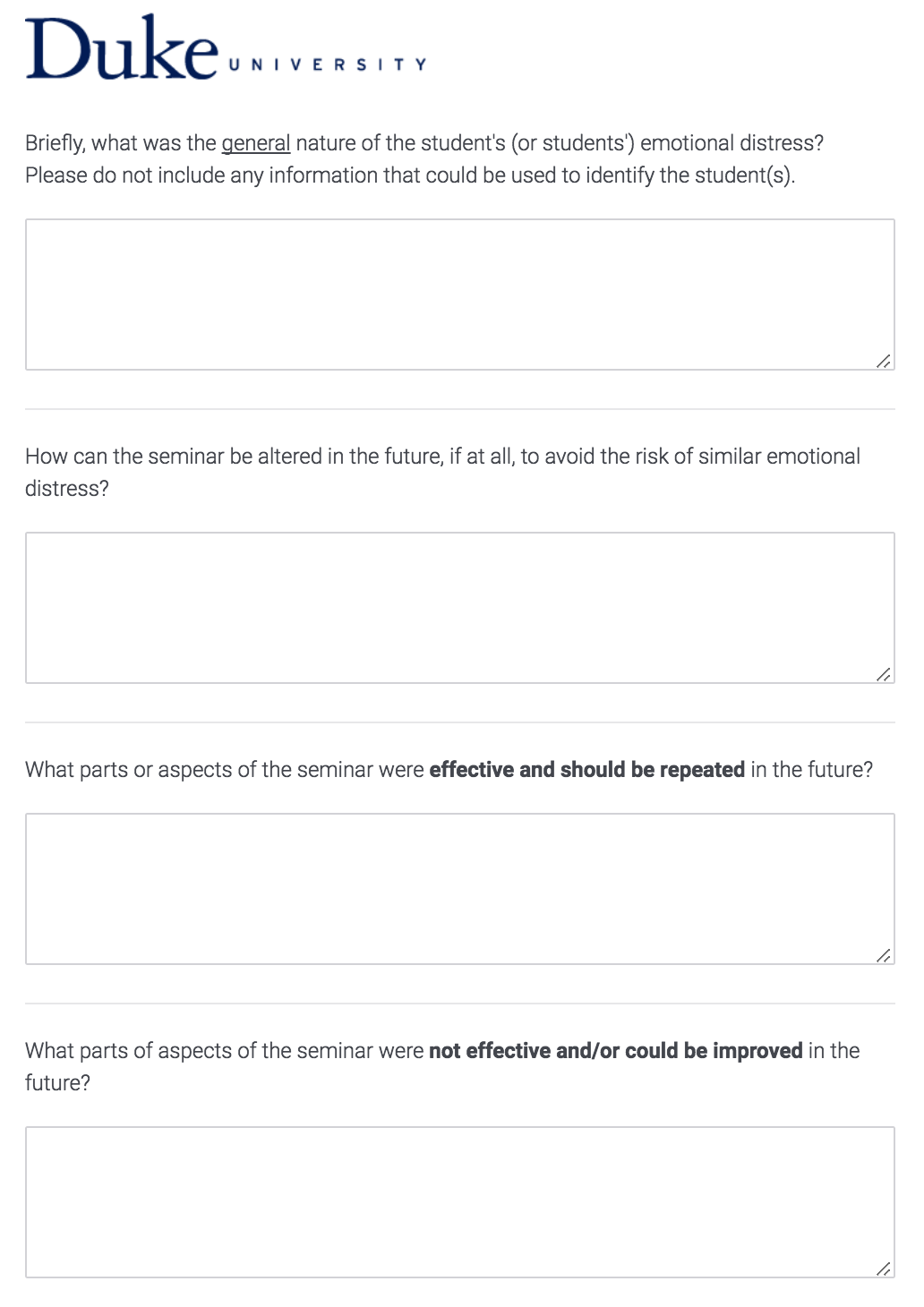

Supplement: Supplementary file 1 — Small Group Facilitator Guide.docxThe Shame Conversation Film.mp4Didactic Slides.pptxSmall Group Discussion Prompts.docxWorkshop Evaluations.docx [file mep_2374-8265.11052-s001.zip › E. Workshop Evaluations.docx]
